# Supplementary material for: Proteomic Identification and Meta-Analysis in Salvia hispanica RNA-Seq de novo Assemblies
Source: Plants (Basel). 2021 Apr 14;10(4):765. doi: 10.3390/plants10040765 (PMC8070742; doi:10.3390/plants10040765)
Supplement: Supplementary file 1 [file plants-10-00765-s001.zip › 20210326-submit-Chia proteogenomics-Supplementary.pdf]

Supplementary Materials for “Proteomic identification and meta-analysis in *Salvia hispanica* RNA-Seq *de novo* assemblies”

Ashwil Klein[1], Lizex H.H. Husselmann[1], Achmat Williams[1], Liam Bell[2], Bret Cooper[3], Brent Ragar[4], David L. Tabb[1,5,6,*]

1 Department of Biotechnology, University of the Western Cape, Bellville, Western Cape, South Africa
2 Centre for Proteomic and Genomic Research, Cape Town, Western Cape, South Africa
3 USDA Agricultural Research Service, Beltsville, MD, United States
4 Departments of Internal Medicine and Pediatrics, Massachusetts General Hospital, Harvard Medical School, Boston, MA, United States
5 Division of Molecular Biology and Human Genetics, Faculty of Medicine and Health Sciences, Stellenbosch University, Cape Town, Western Cape, South Africa
6 Centre for Bioinformatics and Computational Biology, Stellenbosch University, Stellenbosch, South Africa

* dtabb@sun.ac.za

Contents

Supplementary Materials for “Proteomic identification and meta-analysis in *Salvia hispanica* RNA-Seq *de novo* assemblies” 1
Table S1-BUSCO eudicots values for assemblies 3
Table S2-Read mapping rates 4
Table S3-Read mapping concentration 5
Table S4-Six-frame translated sequences and InterProScan / ProteinOrtho matches 5
Table S5-Numbers of distinct peptides identified in Husselmann 2017 6
Table S6-Numbers of distinct peptides identified in Cooper 2021 7
Table S7-Numbers of identifications for Aguilar-Toalá 2019 7
Table S8-Selected QuaMeter IDFree quality metrics 8
Table S9-InterPro signatures for fatty acid desaturases 9
Script S1-Linux shell script to assemble a draft transcriptome 10
Script S2-R Script to produce UpSet plots from ProteinOrtho output 11
Text S1-Husselmann 2017 detailed biochemical methods 13
Plant growth and treatment 13
Protein extraction 13
Sample solubilisation and quantification 13
On-bead HILIC digest 14
Mass Spectrometry 14
Text S2-Cooper 2021 detailed biochemical methods 15
Mass spectrometry 15
Figure S1-Multiple Sequence Alignments of putative fatty acid desaturases 16

Table S1-BUSCO eudicots values for assemblies

BUSCO compares a set of genes expected to appear just once in a set of related species to an assembled transcriptome to evaluate both the completeness and redundancy of the sequences. “plants 9030405” represents the RNA-Seq assembly published with the Wimberley data set. The Peláez data have been split to four separate assemblies since the selected FASTQs represent four different cultivars.

**BUSCO TrimGalore**	Complete single-copy	Complete duplicated	Fragmented	Missing	Sum	Completeness
Gupta	876	1038	152	260	2326	82.29%
Sreedhar	1378	438	142	368	2326	78.07%
Wimberley	881	1266	43	136	2326	92.30%
Peláez Celaya	838	947	159	382	2326	76.74%
Peláez Cualac	854	592	258	622	2326	62.17%
Peláez Jalisco	101	53	114	2058	2326	6.62%
Peláez Veracruz	293	136	253	1644	2326	18.44%
plants 9030405	1549	629	46	102	2326	93.64%
**BUSCO Trimmomatic**	Complete single-copy	Complete duplicated	Fragmented	Missing	Sum	Completeness
Gupta	789	900	256	381	2326	72.61%
Sreedhar	1384	568	97	277	2326	83.92%
Wimberley	701	1524	32	69	2326	95.66%
Peláez Celaya	699	1149	145	333	2326	79.45%
Peláez Cualac	798	770	223	535	2326	67.41%
Peláez Jalisco	101	59	125	2041	2326	6.88%
Peláez Veracruz	309	161	261	1595	2326	20.21%
plants 9030405	1549	629	46	102	2326	93.64%

Table S2-Read mapping rates

The Trinity script "align_and_estimate_abundance" employs Salmon to compare each FASTQ to an assembly to ask the percentage that matches an assembly sequence. The test shown here matched the TrimGalore-filtered reads to an assembly built from them and then matched the TrimGalore-filtered reads to an assembly that was published by Wimberley et alia in *MDPI Plants* (https://doi.org/10.3390/plants9030405).

	FASTQ File	TG Mapping Rate	MDPI Mapping Rate
Gupta	ERR1855102	85.86	78.52
	ERR1855111	87.36	78.23
	ERR1855120	85.89	75.61
	ERR1855129	86.20	75.41
	ERR1855138	82.79	71.96
	ERR1855147	84.76	73.72
	ERR1855155	83.08	77.01
	ERR1855165	84.23	77.68
	ERR1855174	86.83	77.35
	ERR1855184	87.45	77.89
	ERR1855193	88.02	77.65
	ERR1855202	85.26	75.88
	ERR1855210	85.39	64.94
Wimberley	SHHA55_L1	97.96	98.24
	SHHA55_L2	98.15	98.35
	SHHA55_L3	98.07	98.43
	SHHA55_R1	99.73	97.76
	SHHA55_R2	99.68	97.59
	SHHA55_R3	99.72	97.79
Peláez Celaya	SRR6939475	97.13	69.39
	SRR6939476	95.32	70.29
	SRR6939481	97.20	63.30
Sreedhar	SRR824071	98.47	53.83
	SRR824079	98.31	51.50
	SRR824080	98.56	46.65
	SRR824081	98.62	61.35
	SRR824104	98.32	84.55

Table S3-Read mapping concentration

The transcripts produced from a tissue may be concentrated in a small set of genes or spread broadly among many genes. The tables below report the smallest number of transcripts that can account for 25%, 50%, 75% and 100% of all mapped reads (where the assembly derived from those reads is the reference). Each FASTQ pair is reported separately. In Wimberley, the 'L' FASTQs represent leaves while the 'R' FASTQs represent roots. The concentration values illustrate that transcription is focused on a narrower set of transcripts in leaves than in roots.

TGGupta	25%ile	50%ile	75%ile	100%ile
ERR1855102	111	1200	8028	74413
ERR1855111	185	2210	12512	82182
ERR1855120	451	3741	16494	86356
ERR1855129	514	4053	17362	87862
ERR1855138	427	3126	13909	82421
ERR1855147	415	3534	15382	83542
ERR1855155	21	133	1989	61490
ERR1855165	24	175	2876	66606
ERR1855174	462	4047	17154	86657
ERR1855184	328	3318	15382	84865
ERR1855193	160	2472	13266	81254
ERR1855202	514	3903	15897	82706
ERR1855210	36	690	5355	58976

Peláez Celaya	25%ile	50%ile	75%ile	100%ile
SRR6939475	30	511	4839	78291
SRR6939476	47	776	6279	79775
SRR6939481	15	327	4171	93987

Sreedhar	25%ile	50%ile	75%ile	100%ile
SRR824071	10	45	1031	45526
SRR824079	7	33	568	44260
SRR824080	6	19	138	40949
SRR824081	12	76	791	40101
SRR824104	169	733	3347	43929

Wimberley	25%ile	50%ile	75%ile	100%ile
SHHA55_L1	25	168	1990	74045
SHHA55_L2	24	152	1923	72310
SHHA55_L3	18	95	1304	73053
SHHA55_R1	347	1989	9054	100157
SHHA55_R2	343	1986	9180	102144
SHHA55_R3	313	1843	8726	105598

Table S4-Six-frame translated sequences and InterProScan / ProteinOrtho matches

	mRNAs	AA seqs len>100	InterPro hit only	ProteinOrtho hit only	IP and PO hits	No hit
**TGGupta**	145679	216767	71366	2079	49885	93437
**TGPeláez Celaya**	108164	214746	67803	1604	40622	104717
**TGSreedhar**	53127	104952	23167	1362	29304	51119
**TGWimberley**	142899	277842	84787	2441	63020	127594

Associating a novel transcript sequence with molecular function may be attempted by six-frame translation, trimming away sequences below some length threshold, and then seeking orthologs in nearby taxa or sequence motif matches. This table reflects the overlap between orthologs from ProteinOrtho and InterProScan motif matching.

Table S5-Numbers of distinct peptides identified in Husselmann 2017

Each RAW file for Husselmann 2017 represents a separate LC-MS/MS experiment. Each set of tandem mass spectra was identified by database search five different times, using a different protein sequence database to reflect a different assembly.

Distinct Peptides	MDPI Plants	TGGupta	TGPelaez Celaya	TGSreedhar	TGWimberley
Aggregate	7012	7145	5772	6138	6554
1091_B_Caff_acid_3_1.raw	3673	3727	2942	3181	3357
1091_B_Caff_acid_3_2.raw	3175	3258	2556	2735	2892
1091_B_Caff_acid_3_3.raw	4308	4363	3531	3709	4009
1091_B_Caff_acid_3_4.raw	3417	3517	2784	3009	3131
1091_B_Caff_acid_3_5.raw	4500	4525	3659	3880	4188
1091_B_Cnt_1_1.raw	3527	3582	2858	3046	3185
1091_B_Cnt_1_2.raw	3241	3333	2595	2779	2945
1091_B_Cnt_1_3.raw	2231	2262	1826	1910	2011
1091_B_Cnt_1_4.raw	4228	4328	3492	3692	3946
1091_B_Cnt_1_5.raw	3118	3140	2513	2659	2808
1091_B_combo_5_1.raw	3724	3832	2984	3240	3430
1091_B_combo_5_2.raw	3986	4029	3193	3417	3636
1091_B_combo_5_3.raw	3855	3918	3123	3366	3546
1091_B_combo_5_4.raw	4186	4273	3423	3638	3849
1091_B_combo_5_5.raw	4297	4385	3467	3695	3998
1091_B_salt_4_1.raw	3674	3773	3028	3251	3423
1091_B_salt_4_2.raw	3542	3655	2920	3138	3310
1091_B_salt_4_3.raw	3675	3743	2994	3222	3402
1091_B_salt_4_4.raw	3676	3789	3045	3248	3434
1091_B_salt_4_5.raw	3589	3700	2953	3159	3323
1091_Pool_end.raw	3147	3192	2511	2711	2867
1091_Pool_mid1.raw	3096	3125	2475	2656	2803
1091_Pool_mid2.raw	2782	2832	2206	2391	2523
1091_Pool_start.raw	2713	2817	2167	2354	2442
1091_W_Cnt_2_1.raw	3114	3227	2512	2706	2865
1091_W_Cnt_2_2.raw	3179	3187	2530	2703	2893
1091_W_Cnt_2_3.raw	3006	3090	2408	2580	2767
1091_W_Cnt_2_4.raw	3126	3176	2504	2674	2837
1091_W_Cnt_2_5.raw	2334	2352	1752	1923	2071

Table S6-Numbers of distinct peptides identified in Cooper 2021

The same set of five protein sequences databases were used to identify the different LC-MS/MS experiments of Cooper 2021.

DISTINCT PEPTIDES	MDPI Plants	TGGupta	TGPelaez Celaya	TGSreedhar	TGWimberley
Aggregate	31327	32923	27882	31911	31666
LD-QC-LE_201202_CooperB_BC_S1cot 500ng_FTIT.raw	8461	9016	7406	8154	8003
LD-QC-LE_201202_CooperB_BC_S2hyp 500ng_FTIT.raw	12663	13383	11285	12834	12709
LD-QC-LE_201202_CooperB_BC_S3root 500ng_FTIT.raw	10678	10682	9332	10575	11167
LD-QC-LE_201202_CooperB_BC_S4seed 500ng_FTIT.raw	4275	5528	5338	6122	4213
LD-QC-LE_210104_CooperB_BC_S1cot_F1.raw	2673	2977	2312	2641	2622
LD-QC-LE_210104_CooperB_BC_S1cot_F2.raw	4260	4595	3784	4236	4014
LD-QC-LE_210104_CooperB_BC_S1cot_F3.raw	7354	7778	6629	7089	7084
LD-QC-LE_210104_CooperB_BC_S1cot_F4.raw	7851	8225	6818	7501	7409
LD-QC-LE_210104_CooperB_BC_S2hyp_F1.raw	5301	5705	4810	5425	5393
LD-QC-LE_210104_CooperB_BC_S2hyp_F2.raw	7274	7823	6426	7333	7327
LD-QC-LE_210104_CooperB_BC_S2hyp_F3.raw	10814	11337	9702	10963	10968
LD-QC-LE_210104_CooperB_BC_S2hyp_F4.raw	11174	11706	10043	11186	11157
LD-QC-LE_210104_CooperB_BC_S3root_F1.raw	6187	6253	5526	6076	6467
LD-QC-LE_210104_CooperB_BC_S3root_F2.raw	6218	6468	5466	6233	6573
LD-QC-LE_210104_CooperB_BC_S3root_F3.raw	9309	9304	8258	9179	9816
LD-QC-LE_210104_CooperB_BC_S3root_F4.raw	8979	8785	7715	8794	9291

Table S7-Numbers of identifications for Aguilar-Toalá 2019

Because enzymatic specificity was more ambiguous for the alcalase / flavourzyme combination employed by Aguilar-Toalá, MS-GF+ rather than MSFragger was used for the search.

010_2019_11_016_R_Liceaga AguilarToala_less3kD.raw	MDPI Plants	TGGupta	TGPelaez Celaya	TGSreedhar	TGWimberley
Distinct peptides	200	697	620	445	118
Distinct matches	244	880	787	532	140
Confident PSMs	267	1068	945	597	144

Table S8-Selected QuaMeter IDFree quality metrics

A selection of metrics from QuaMeter IDFree characterize the LC-MS/MS experiments. The extremely high sensitivity of the Orbitrap Fusion Lumos Tribrid results from its scan rate, surpassing 20 MS/MS per second (MS2-Freq-Max column) in some experiments. The charge state distribution for Aguilar-Toalá 2019 hints that the "less3kD" sample contains the most peptide information (MS2-PrecZ-1, 2, and 3). The stability of metrics among the Husselmann 2017 set reflects well upon the quality processes in place at the core facility.

Husselmann 2017	RT-Duration	MS1-Count	MS1-Freq-Max	MS2-Count	MS2-Freq-Max	MS2-PrecZ-1	MS2-PrecZ-2	MS2-PrecZ-3
1091_B_Caff_acid_3_1	4920.18	7438	2.11652	34861	7.39957	0%	14%	8%
1091_B_Caff_acid_3_2	4920	7861	2.4301	33417	7.34451	0%	14%	8%
1091_B_Caff_acid_3_3	4919.98	7386	1.90668	35095	7.39987	0%	15%	8%
1091_B_Caff_acid_3_4	4919.94	7890	2.39668	33304	7.35984	0%	15%	9%
1091_B_Caff_acid_3_5	4920.16	7507	2.09536	34816	7.427	0%	15%	9%
1091_B_Cnt_1_1	4920.05	7339	1.93106	35173	7.39054	0%	14%	8%
1091_B_Cnt_1_2	4920.02	7524	1.99519	34652	7.37577	0%	14%	7%
1091_B_Cnt_1_3	4919.96	7426	1.92851	34813	7.34288	0%	12%	7%
1091_B_Cnt_1_4	4920.19	7421	1.98549	35031	7.41382	0%	14%	9%
1091_B_Cnt_1_5	4919.94	7426	2.09343	34858	7.36589	0%	13%	8%
1091_B_combo_5_1	4919.98	7417	2.12108	34921	7.39972	0%	14%	8%
1091_B_combo_5_2	4920.01	7456	2.06621	34839	7.39999	0%	14%	8%
1091_B_combo_5_3	4920.2	7894	2.44939	33361	7.39102	0%	15%	9%
1091_B_combo_5_4	4920.17	7578	2.24659	34416	7.39971	0%	14%	9%
1091_B_combo_5_5	4920.12	7782	2.55125	33769	7.3998	0%	15%	9%
1091_B_salt_4_1	4920.17	7479	2.16128	34653	7.38295	0%	14%	8%
1091_B_salt_4_2	4920.15	7615	2.24777	34243	7.36465	0%	14%	8%
1091_B_salt_4_3	4919.97	7479	2.10313	34703	7.38023	0%	14%	8%
1091_B_salt_4_4	4920	7733	2.19762	33917	7.37981	0%	14%	8%
1091_B_salt_4_5	4920.22	7562	2.16163	34497	7.35926	0%	14%	8%
1091_Pool_end	4920.16	7476	2.00361	34723	7.38292	0%	14%	7%
1091_Pool_mid1	4920.02	7510	2.09855	34488	7.37881	0%	15%	8%
1091_Pool_mid2	4920	7354	1.88255	35016	7.36041	0%	14%	7%
1091_Pool_start	4920.17	7832	2.41814	33495	7.33107	0%	15%	7%
1091_W_Cnt_2_1	4920.02	7439	1.98228	34751	7.36658	0%	13%	8%
1091_W_Cnt_2_2	4920.02	7437	2.01682	34923	7.36664	0%	13%	7%
1091_W_Cnt_2_3	4920.01	7417	1.96865	34881	7.381	0%	13%	8%
1091_W_Cnt_2_4	4919.98	7508	2.26248	34565	7.3974	0%	13%	8%
1091_W_Cnt_2_5	4920.04	7447	2.07891	34682	7.39127	0%	12%	7%
Aguilar-Toalá 2019	RT-Duration	MS1-Count	MS1-Freq-Max	MS2-Count	MS2-Freq-Max	MS2-PrecZ-1	MS2-PrecZ-2	MS2-PrecZ-3
010_2019_11_016_R_Liceaga_AguilarToala_less3kD	5399.84	5950	2.40174	44208	10.907	29%	30%	5%
010_2019_11_016_R_Liceaga_AguilarToala_FII-2	5399.93	10583	2.34621	19346	10.731	42%	2%	1%
010_2019_11_016_R_Liceaga_AguilarToala_FIV-2	5399.78	10835	2.34513	17754	10.7317	39%	1%	1%
Cooper 2021	RT-Duration	MS1-Count	MS1-Freq-Max	MS2-Count	MS2-Freq-Max	MS2-PrecZ-1	MS2-PrecZ-2	MS2-PrecZ-3
LD-QC-LE_201202_CooperB_BC_S1cot_500ng_FTIT	7199.33	15724	2.53563	65234	19.7562	0%	48%	43%
LD-QC-LE_201202_CooperB_BC_S2hyp_500ng_FTIT	7199.34	14713	2.5	78206	20.4826	0%	46%	43%
LD-QC-LE_201202_CooperB_BC_S3root_500ng_FTIT	7199.62	14889	2.50097	74304	20.0903	0%	41%	44%
LD-QC-LE_201202_CooperB_BC_S4seed_500ng_FTIT	7199.55	16040	2.5492	65730	18.3946	0%	36%	47%
LD-QC-LE_210104_CooperB_BC_S1cot_F1	7199.47	16666	2.52017	15322	8.41454	0%	62%	32%
LD-QC-LE_210104_CooperB_BC_S1cot_F2	7199.49	16812	2.52892	32148	13.3662	0%	57%	37%
LD-QC-LE_210104_CooperB_BC_S1cot_F3	7199.32	15788	2.55364	64057	20.5878	0%	47%	43%
LD-QC-LE_210104_CooperB_BC_S1cot_F4	7199.59	15849	2.56758	59176	20.2781	0%	46%	44%
LD-QC-LE_210104_CooperB_BC_S2hyp_F1	7199.56	16704	2.49642	26062	12.9808	0%	50%	38%
LD-QC-LE_210104_CooperB_BC_S2hyp_F2	7199.32	16313	2.48667	46672	15.9442	0%	51%	39%
LD-QC-LE_210104_CooperB_BC_S2hyp_F3	7199.67	15141	2.4687	74312	21.0491	0%	44%	45%
LD-QC-LE_210104_CooperB_BC_S2hyp_F4	7199.34	15025	2.50752	70098	20.8595	0%	42%	45%
LD-QC-LE_210104_CooperB_BC_S3root_F1	7199.56	16705	2.48625	27785	13.5012	0%	38%	44%
LD-QC-LE_210104_CooperB_BC_S3root_F2	7199.28	16606	2.48019	39475	15.0207	0%	48%	41%
LD-QC-LE_210104_CooperB_BC_S3root_F3	7199.62	15137	2.48154	73137	20.5162	0%	41%	46%
LD-QC-LE_210104_CooperB_BC_S3root_F4	7199.26	15167	2.50098	66167	20.5106	0%	35%	45%

Table S9-InterPro signatures for fatty acid desaturases

Investigating the fatty acid desaturases that enable PUFA production in chia led us to create a list of current “integrated” signatures at InterPro that represent this class of enzymes. The signatures vary widely in the number of transcripts they match across the translated TrimGalore assemblies.

	**Gupta**	**Peláez Celaya**	**Sreedhar**	**Wimberley**	**Sum**
**IPR001522**	0	2	0	0	2
**IPR005067**	83	24	41	113	261
**IPR005803**	19	5	8	12	44
**IPR005804**	37	30	29	50	146
**IPR012171**	9	8	6	6	29
**IPR021863**	15	7	11	26	59
**Sum**	163	76	95	207	

Script S1-Linux shell script to assemble a draft transcriptome

This script shows the set of Linux commands used to download, trim, and assemble a transcriptome, using Peláez Celaya as an example.

```
# Acquire FASTQs via NCBI SRA Toolkit
prefetch SRR6939475 SRR6939476 SRR6939481
fastq-dump --defline-seq '@$sn[_$rn]/$ri' --defline-qual '+'
      --split-files SRR6939475 SRR6939476 SRR6939481

# Employ TrimGalore to remove adapters and lowest-quality reads
/usr/local/bin/TrimGalore-0.6.6/trim_galore --cores 6 --length 25 --paired
      --output_dir galore -q 5 --stringency 1 -e 0.1 SRR6939475_1.fastq
      SRR6939475_2.fastq
/usr/local/bin/TrimGalore-0.6.6/trim_galore --cores 6 --length 25 --paired
      --output_dir galore -q 5 --stringency 1 -e 0.1 SRR6939476_1.fastq
      SRR6939476_2.fastq
/usr/local/bin/TrimGalore-0.6.6/trim_galore --cores 6 --length 25 --paired
      --output_dir galore -q 5 --stringency 1 -e 0.1 SRR6939481_1.fastq
      SRR6939481_2.fastq

# Establish locations of Trinity support libraries
export TRINITY_HOME=/usr/local/bin/trinityrnaseq-v2.11.0
export PATH=$PATH:/usr/local/bin/salmon-1.3.0_linux_x86_64/bin
export PATH=$PATH:/usr/local/bin/bowtie2-2.4.2-linux-x86_64
export PATH=$PATH:/usr/local/bin/jellyfish-2.3.0

# Produce Trinity assembly, limiting RAM usage to 80 GB and using 6 cores
/usr/local/bin/trinityrnaseq-v2.11.0/Trinity --seqType fq --max_memory 80G
      --left SRR6939475_1_val_1.fq.gz,SRR6939476_1_val_1.fq.gz,
      SRR6939481_1_val_1.fq.gz --right SRR6939475_2_val_2.fq.gz,
      SRR6939476_2_val_2.fq.gz,SRR6939481_2_val_2.fq.gz --CPU 6

# Evaluate read mapping in Salmon to the created FASTA
/usr/local/bin/trinityrnaseq-v2.11.0/util/align_and_estimate_abundance.pl
      --transcripts TGPelaezCelaya.fna --est_method salmon --trinity_mode
      --prep_reference
/usr/local/bin/trinityrnaseq-v2.11.0/util/align_and_estimate_abundance.pl
      --transcripts TGPelaezCelaya.fna --est_method salmon --trinity_mode
      --seqType fq --samples_file salmon_PelaezCelaya.txt
/usr/local/bin/salmon-1.3.0_linux_x86_64/bin/salmon quantmerge --quants
      SRR6939475 SRR6939476 SRR6939481 --names SRR6939475 SRR6939476
      SRR6939481 -o Salmon-TGPelaezCelaya.tsv
```

Script S2-R Script to produce UpSet plots from ProteinOrtho output

ProteinOrtho's HTML output is easy to navigate for detailed information on individual sets of orthologs. Visualizing the intersections among multiple transcriptomes at a higher level is greatly aided by the creation of UpSet plots.

```
library(UpSetR)
# Compare the different transcriptomes within TG assemblies
PO <- read.table("TG.proteinortho.tsv", header=FALSE, sep="\t",skip=1);
names(PO) <- c("SpeciesCount","Genes","AlgConn","TGGupta",
      "TGPelaezCelaya","TGSreedhar","TGWimberley","plants9030405")
TGGupta = which(PO$TGGupta!="*")
TGPelaezCelaya = which(PO$TGPelaezCelaya!="*")
TGSreedhar = which(PO$TGSreedhar!="*")
TGWimberley = which(PO$TGWimberley!="*")
Plants = which(PO$plants9030405!="*")
AllList <- list(TGGupta,TGPelaezCelaya,TGSreedhar,TGWimberley,Plants)
names(AllList)= c("TGGupta","TGPelaezCelaya","TGSreedhar","TGWimberley",
      "Plants")
upset(fromList(AllList), order.by= "freq")

# Figure 3
upset(fromList(AllList), order.by= "freq", nintersects=12,
show.numbers="no")

# Compare the translations among species for Gupta
PO <- read.table("TaxTGGupta.proteinortho.tsv", header=FALSE,
      sep="\t",skip=1);
NameVector = c("G.aurea","D.hygrometricum","H.impetiginosus",
      "S.splendens","S.asiatica","P.japonicum","A.thaliana","E.guttata",
      "S.indicum","O.europaea","TGGupta")
names(PO) <- c("SpeciesCount","Genes","AlgConn",NameVector)
PO <- subset(PO, SpeciesCount>1)
G.aurea = which(PO$G.aurea!="*")
D.hygrometricum = which(PO$D.hygrometricum!="*")
H.impetiginosus = which(PO$H.impetiginosus!="*")
S.splendens = which(PO$S.splendens!="*")
S.asiatica = which(PO$S.asiatica!="*")
P.japonicum = which(PO$P.japonicum!="*")
A.thaliana = which(PO$A.thaliana!="*")
E.guttata = which(PO$E.guttata!="*")
S.indicum = which(PO$S.indicum!="*")
O.europaea = which(PO$O.europaea!="*")
TGGupta = which(PO$TGGupta!="*")
```

```
AllList <- list(G.aurea, D.hygrometricum, H.impetiginosus, S.splendens,
      S.asiatica, P.japonicum, A.thaliana, E.guttata, S.indicum,
      O.europaea,TGGupta)
names(AllList)=NameVector
upset(fromList(AllList), order.by= "freq",nsets=11)

# Figure 4
upset(fromList(AllList), order.by="freq",nsets=11,
      nintersects=12,show.numbers=F)
```

Text S1-Husselmann 2017 detailed biochemical methods

Plant growth and treatment

Chia (*Salvia hispanica L.*) seeds (WSG (white seed genotype) and BSG (black seed genotype)) were germinated on moist filter paper in a dark environment for 2-3 days. The germinated seedlings were transplanted (1 per pot) in a moist (distilled water) Promix growth medium (Stodels Garden Centre, South Africa) and allowed to grow in a growth room on a 16 hours light/8 hours dark cycle at 25°C until the collar of the second true leaves were visible. At this stage seedlings were supplemented with 1x nutrient solution mixture [1mM K2SO4 (Sigma Aldrich), 1 µM ZnSO4 (Sigma Aldrich), 5 mM CaCl2 (Sigma Aldrich), 5 µM MnSO4 (Sigma Aldrich), 10 mM NH4NO3 (Sigma Aldrich), 5 mM KNO3 (Sigma Aldrich), 5 µM H3BO3 (Sigma Aldrich), 1 mM K2HPO4 (Sigma Aldrich) buffer at pH 6.4, 10 mM 4-(2-hydroxyethyl)-1piperazineethanesulfonic acid (HEPES) (Sigma Aldrich) at pH 6.4, 100 µM Fe-NaEDTA (Sigma Aldrich), 2 µM Na2MoO4 (Sigma Aldrich), 1 µM CoSO4 (Sigma Aldrich), 1 µM CuSO4 (Sigma Aldrich) and 2 mM MgSO4 (Sigma Aldrich)] containing 100 µM caffeic acid (Sigma Aldrich), 100 mM NaCl (Sigma Aldrich), and a combination of 100 µM caffeic acid and 100 mM NaCl. Treatments were applied to each plant directly at the base of the stem at 2 days intervals for a period of 21 days.

Protein extraction

Total leaf protein for each genotype was extracted using the phenol/SDS method as previously described by Wang et al. (https://doi.org/10.1002/elps.200500722) with slight modifications. Protein extracts for each treatment were obtained by homogenizing 0.25 g of freshly ground leaf tissue in 10% TCA/acetone (Sigma Aldrich, Merck). The homogenates were centrifuged at 16,000 x g for 10 min at 4°C and the supernatant decanted. The pellet was washed once with methanolic ammonium acetate (0.1 M ammonium acetate (Sigma Aldrich) dissolved in 80% methanol (Merck) and 80% acetone and the supernatant decanted after each centrifugation (16,000 x g for 5 min) step. The pellet was dried at room temperature and re-suspended in 0.8 mL dense sodium dodecyl sulfate (SDS) (Bio-Rad) buffer (2% SDS, 0.1 M Tris-HCl (Sigma Aldrich), pH 8.0, 1 mM phenylmethylsulfonyl fluoride (PMSF) (Sigma Aldrich), 5% 2-mercaptoethanol (Merck), 30% sucrose (Sigma Aldrich),) and 0.8 ml phenol (Tris-buffered, pH 8.0; Sigma Aldrich) in a 2 ml eppendorf tube. The mixture was thoroughly vortexed for 3 min and the phenol phase was partitioned by centrifugation at 16,000 x g for 10 min at 4°C. The upper phase (phenol) was transferred to fresh Eppendorf tubes (0.5 ml for 2 ml tube). The extraction process was repeated, and phenol fractions were pooled. Proteins were precipitated overnight with 3 volumes of pre-cooled methanolic ammonium acetate (0.1 M ammonium acetate dissolved in 80% menthanol (Merck)). Precipitated proteins were recovered at 16,000 x g for 10 min (4°C), washed with cold methanolic ammonium acetate and 80% acetone. The protein pellets were air dried at room temperature and analysed at the Centre for Proteomic and Genomic Research (CPGR).

Sample solubilisation and quantification

Protein pellets were solubilised by resuspending in 50 mM triethylammonium bicarbonate (TEAB; Sigma T7408) 2% Sodium dodecyl sulfate (SDS; Sigma 71736) and placing at 95˚C for five minutes. Thereafter samples were clarified by centrifugation at 10000 x g for five

minutes. Quantification was performed using the QuantiPro BCA assay kit (Sigma QPBCA) according to the manufacturer's instructions.

On-bead HILIC digest

In preparation for the HILIC magnetic bead workflow, beads (ReSyn Biosciences, MR-HLC002) were aliquoted into a new tube and the shipping solution removed. Beads were then washed with 250 µl wash buffer (15% ACN, 100 mM Ammonium acetate (Sigma 14267) pH 4.5) for one minute. This was repeated once. The beads were then resuspended in loading buffer (30% ACN, 200 mM Ammonium acetate pH 4.5). The rest of the process described hereafter was performed using a Hamilton MassSTAR robotics liquid handler (Hamilton, Switzerland). A total of 50 µg of protein from each sample was transferred to a protein LoBind plate (Merck, 0030504.100). Protein was reduced with tris (2-carboxyethyl) phosphine (TCEP; Sigma 646547) which was added to a final concentration of 10 mM TCEP and incubated at 60˚C for one hour. Samples were cooled to room temperature and then alkylated with methylmethanethiosulphonate (MMTS; Sigma 208795) which was added to a final concentration of 10 mM MMTS and incubated at room temperature for 15 minutes. HILIC magnetic beads were added at an equal volume to that of the sample and a ratio of 5:1 total protein. The plate was then incubated at room temperature on the shaker at 900RPM for 30 minutes for binding of protein to beads. After binding, the beads were washed four times with 500 µl of 95% ACN for one minute. For digestion Trypsin (Promega PRV5111), made up in 50 mM TEAB was added at a ratio of 1:10 total protein and the plate was incubated at 37˚C on the shaker for four hours. After digestion, the supernatant containing peptides was removed and dried down. Samples were resuspended in 0.1% trifluoroacetic acid (TFA, Sigma T6508) prior to cleanup by Zip-Tip (Sigma Z720070). Thereafter, samples were dried down once more and then resuspended in LC-MS loading buffer: 0.1% FA, 2.5% ACN.

Mass Spectrometry

LC-MS analysis was conducted with a Q-Exactive quadrupole-Orbitrap mass spectrometer (Thermo Fisher Scientific, USA) coupled with a Dionex Ultimate 3000 nano-HPLC system. Peptides were dissolved in 0.1% Formic Acid (FA; Sigma 56302), 2% Acetonitrile (ACN; Burdick & Jackson BJLC015CS) and loaded on a C18 trap column (300 µm x 5 mm x 5 µm) at 3.5% solvent B and a flow rate of 5 µL/min and washed for four minutes. The trap is then switched in-line with the analytical column for loading of peptides for 26 minutes. Thereafter the trap is switched offline to prevent loading of hydrophobic contaminants onto the analytical column. Chromatographic separation was performed with a PepAcclaim C18 column (75 µm x 25 cm x 2 µm) as described below. The solvent system employed was solvent A: LC water (Burdick and Jackson BJLC365); 0.1% FA and solvent B: ACN, 0.1% FA. The multi-step gradient for peptide separation was generated as 300 nL/min as follows: time change 6 min, gradient change: 3.5-9% Solvent B, time change 45.5 min, gradient change 9-24.6% Solvent B, time change 2 min, gradient change 24.6-38.7% Solvent B, time change 2.1 min, gradient change 38.7-52.8% Solvent B, time change 0.4 min, gradient change 52.8-85.4%. The gradient was then held at 85.4% solvent B for 10 minutes before returning it to 3.5% solvent B for 15 minutes to condition the column. The mass spectrometer was operated in positive ion mode with a capillary temperature of 320 degrees C. The applied electrospray voltage was 1.95 kV.

Text S2-Cooper 2021 detailed biochemical methods

Heirloom *Salvia columbariae* were obtained from Seeds of Change (Rancho Dominquez, California, U.S.A.). The seeds were sown in soil and placed on indoor light-carts. Seedlings were harvested 5 days later and dissected at the root, hypocotyl, and cotyledon junctions. One gram each of roots, hypocotyls, and cotyledons, and half a gram of ungerminated seeds were ground to a powder with a mortar and pestle and further triturated in 2 volumes of 100 mM Tris pH 8.5, 5 mM dithiothreitol, 1 mM EDTA, and 0.5% dodecyl beta maltoside. The extract was centrifuged for 5,000 x g for 10 minutes. The supernatant was reserved while the pellet was washed one time in 2 volumes of 100 mM Tris pH 8.5, 5 mM dithiothreitol, 1 mM EDTA, resuspended in the same and sonicated 2 times for 30 seconds each time, and then centrifuged 5,000 x g for 10 minutes. The supernatant from the sonicated pellet was combined with the prior supernatant and proteins were precipitated in acetone/25% trichloroacetic acid and isolated by centrifugation at 15,000 x g. The proteins were washed in acetone 2 times, dried, and resuspended in 8 M urea/100 mM Tris pH 8.5/0.1% dodecyl beta maltoside. The protein concentration was determined by bicinchoninic acid assay (Pierce, Rockford, IL).

Proteins (~100 µg) were reduced in Tris(2-carboxyethyl)phosphine, carboxyamidomethylated with iodoacetamide, and digested overnight at 37 °C with immobilized TPCK-Trypsin (Thermo Fisher Scientific, Waltham, MA, USA). The digested peptides were purified by reverse phase chromatography using SPEC PT C18 columns (Agilent Technologies, Santa Clara, CA, USA) and their concentrations measured with the Pierce Quantitative Colorimetric Peptide Assay (Thermo Fisher Scientific).

Mass spectrometry

Mass spectrometry was performed at the Mass Spectrometry and Proteomics Facility at the Johns Hopkins School of Medicine. Peptides from leaf cotyledons, hypocotyls, roots, and seeds (500 ng) were analyzed by mass spectrometry (below). Subsequent analysis revealed that seed samples were not as complex as the leaf cotyledon, hypocotyl, and root samples. To obtain deeper analysis of the leaf cotyledon, hypocotyl, and root samples, thirteen micrograms of peptides were step-fractioned on an Oasis plate (Waters, Milford, MA) by eluting into 4 fractions (elution with 10mM TEAB two times followed by 5% ACN [the three elutions were combined], followed by separate 10%, 25%, and 75% ACN elutions). Fractioned or non-fractioned peptides (500 ng) were separated on a 75 µm (inner diameter) fused silica capillary C18 column using a 0-60% ACN/0.1% formic acid gradient over 90 minutes at 300 nL per minute. Peptides were electrosprayed at 2.5 kV into an Orbitrap Fusion Lumos Tribrid mass spectrometer (Thermo Fisher Scientific) operating in data-dependent mode with positive polarity and using Easy-IC internal mass calibration. Quadrupole isolation was enabled. Survey scans were recorded in the Orbitrap at 120,000 resolution over a mass range of 400-1600 m/z. The instrument was operated in Top Speed mode with a cycle time of 3 seconds. The automatic gain control (AGC) target was set to 600,000 and the maximum injection time was set to 50 milliseconds (msec). The most abundant precursor ions (intensity threshold 50,000) were fragmented by high-energy collision-induced dissociation (30% energy) and fragment ions were detected in the linear ion trap (AGC 20,000, 35 msec maximum injection). Analyzed precursors were dynamically excluded for 20 seconds.

Figure S1-Multiple Sequence Alignments of putative fatty acid desaturases

The next ten images all represent multiple sequence alignment of transcripts, where a particular transcript is observed in all four of the assemblies and the sequences match IPR005804, a fatty acid desaturase enzyme motif. *The sequences underlying each alignment are available in a Zip file that is part of Supplementary Information at the journal website.*

Alignment 1: These translated transcripts match the cloned sequences for KX610643 and KX610644 produced by Yufei Xue *et al*. (10.1007/s11738-017-2390-0) to a high degree of homology, suggesting that these sequences correspond to FAD2. The amino acid sequences from these four transcriptome assemblies are nearly identical, with variants only in the third and penultimate rows of the alignment. BLAST recognizes this sequence as having homology to A0A1Z1EC55_9LAMI: “Fatty acid desaturase 2 isoform 2.”

```
CLUSTAL O(1.2.4) multiple sequence alignment


WIMBERLEY_DN689_c0_g1_i3      MGAGGRMSVPPAEKAAKSDIVQRVPHTKPPFTLGDIKKAIPPHCFKRSIPRSFSYVVYDL	60
GUPTA_DN4241_c0_g1_i2         MGAGGRMSVPPAEKAAKSDIVQRVPHTKPPFTLGDIKKAIPPHCFKRSIPRSFSYVVYDL	60
SREEDHAR_DN157_c0_g1_i10      MGAGGRMSVPPAEKAAKSDIVQRVPHTKPPFTLGDIKKAIPPHCFKRSIPRSFSYVVYDL	60
PELAEZ_DN4790_c0_g1_i3        MGAGGRMSVPPAEKAAKSDIVQRVPHTKPPFTLGDIKKAIPPHCFKRSIPRSFSYVVYDL	60
                              ************************************************************

WIMBERLEY_DN689_c0_g1_i3      VFASLFYYVATNYIHQLPHPLSYPAWILYGICQGCILTGVWVIAHECGHHAFSDYQWLDD	120
GUPTA_DN4241_c0_g1_i2         VFASLFYYVATNYIHQLPHPLSYPAWILYGICQGCILTGVWVIAHECGHHAFSDYQWLDD	120
SREEDHAR_DN157_c0_g1_i10      VFASLFYYVATNYIHQLPHPLSYPAWILYGICQGCILTGVWVIAHECGHHAFSDYQWLDD	120
PELAEZ_DN4790_c0_g1_i3        VFASLFYYVATNYIHQLPHPLSYPAWILYGICQGCILTGVWVIAHECGHHAFSDYQWLDD	120
                              ************************************************************

WIMBERLEY_DN689_c0_g1_i3      TVGLILHSFLLVPFFSWKYSHRRHHSNTGSLERDEVFVPKVKSGVSWTAKYMNNPPGRLI	180
GUPTA_DN4241_c0_g1_i2         TVGLILHSFLLVPFFSWKYSHRRHHSNTGSLERDEVFVPKVKTGVSWAAKYMNNPPGRLI	180
SREEDHAR_DN157_c0_g1_i10      TVGLILHSFLLVPFFSWKYSHRRHHSNTGSLERDEVFVPKVKTGVSWAAKYMNNPPGRLI	180
PELAEZ_DN4790_c0_g1_i3        TVGLILHSFLLVPFFSWKYSHRRHHSNTGSLERDEVFVPKVKTGVSWAAKYMNNPPGRLI	180
                              ******************************************:****:************

WIMBERLEY_DN689_c0_g1_i3      TLVVQLTLGWPLYLMFNVSGRPYDRFACHFDPNSPIYSDRERAQIFISDAGILAVTYGLY	240
GUPTA_DN4241_c0_g1_i2         TLVVQLTLGWPLYLMFNVSGRPYDRFACHFDPNSPIYSDRERAQIFISDAGILAVTYGLY	240
SREEDHAR_DN157_c0_g1_i10      TLVVQLTLGWPLYLMFNVSGRPYDRFACHFDPNSPIYSDRERAQIFISDAGILAVTYGLY	240
PELAEZ_DN4790_c0_g1_i3        TLVVQLTLGWPLYLMFNVSGRPYDRFACHFDPNSPIYSDRERAQIFISDAGILAVTYGLY	240
                              ************************************************************

WIMBERLEY_DN689_c0_g1_i3      RLSVAKGLAWVLCVYGGPLLVVNGFLVLITFLQHTHPSLPHYDSSEWDWLRGALSTVDRD	300
GUPTA_DN4241_c0_g1_i2         RLSVAKGLAWVLCVYGGPLLVVNGFLVLITFLQHTHPSLPHYDSSEWDWLRGALSTVDRD	300
SREEDHAR_DN157_c0_g1_i10      RLSVAKGLAWVLCVYGGPLLVVNGFLVLITFLQHTHPSLPHYDSSEWDWLRGALSTVDRD	300
PELAEZ_DN4790_c0_g1_i3        RLSVAKGLAWVLCVYGGPLLVVNGFLVLITFLQHTHPSLPHYDSSEWDWLRGALSTVDRD	300
                              ************************************************************

WIMBERLEY_DN689_c0_g1_i3      YGILNTVFHNITDTHVAHHLFSTMPHYHAMEATKAIKPILGKYYQFDGTPVFKAMFREVK	360
GUPTA_DN4241_c0_g1_i2         YGILNTVFHNITDTHVAHHLFSTMPHYHAMEATKVIKPILGKYYQFDGTPVFKAMFREVK	360
SREEDHAR_DN157_c0_g1_i10      YGILNTVFHNITDTHVAHHLFSTMPHYHAMEATKVIKPILGKYYQFDGTPVFKAMFREVK	360
PELAEZ_DN4790_c0_g1_i3        YGILNTVFHNITDTHVAHHLFSTMPHYHAMEATKAIKPILGKYYQFDGTPVFKAMFREVK	360
                              **********************************.*************************

WIMBERLEY_DN689_c0_g1_i3      ECIYVEPDEGEENKGVFWYNNKL	383
GUPTA_DN4241_c0_g1_i2         ECIYVEPDEGEENKGVFWYNNKL	383
SREEDHAR_DN157_c0_g1_i10      ECIYVEPDEGEENKGVFWYNNKL	383
PELAEZ_DN4790_c0_g1_i3        ECIYVEPDEGEENKGVFWYNNKL	383
                              ***********************
```

Alignment 2: These sequences bear high homology to A0A4D8Y632_SALSN: “Sphingolipid delta(4)-desaturase DES1-like,” where SALSN is the abbreviation for *Salvia splendens*.

```
CLUSTAL O(1.2.4) multiple sequence alignment


SREEDHAR_DN8237_c0_g2_i1        MGFEGKEGAMATDFFWSYTDEPHASRRRQILSQYPQIKQLFGPDPFAFLKIAAVVLLQLW    60
GUPTA_DN18238_c0_g1_i2          ---------MATDFFWSYTDEPHASRRRQILSQYPQIKQLFGPDPFAFLKIAAVVLLQLW    51
PELAEZ_DN35633_c0_g1_i1         MGFEGKEGAMATDFFWSYTDEPHASRRRQILSQYPQIKQLFGPDPFAFLKIAAVVLLQLW    60
WIMBERLEY_DN10949_c0_g1_i1      ---------MATDFFWSYTDEPHASRRRQILSQYPQIKQLFGPDPFAFLKIAAVVLLQLW    51
                                         ***************************************************

SREEDHAR_DN8237_c0_g2_i1        TATFLHNASWVKILIVAYFFGSFLNHNLFLAIHELSHNLAFLTPVYNRWLGIFANLPIGV    120
GUPTA_DN18238_c0_g1_i2          TATFLHNASWVKILIVAYFFGSFLNHNLFLAIHELSHNLAFLTPVYNRWLGIFANLPIGV    111
PELAEZ_DN35633_c0_g1_i1         TATFLHNASWVKILIVAYFFGSFLNHNLFLAIHELSHNLAFLTPVYNRWLGIFANLPIGV    120
WIMBERLEY_DN10949_c0_g1_i1      TATFLHNASWVKILIVAYFFGSFLNHNLFLAIHELSHNLAFLTPVYNRWLGIFANLPIGV    111
                                ************************************************************

SREEDHAR_DN8237_c0_g2_i1        PMSVTFQKYHLEHHRYQGVDGLDMDVPSLTEAKVVRNTVSKSIWVVLQLFFYALRPLFLK    180
GUPTA_DN18238_c0_g1_i2          PMSVTFQKYHLEHHRYQGVDGLDMDVPSLTEAKVVRNTVSKSIWVVLQLFFYALRPLFLK    171
PELAEZ_DN35633_c0_g1_i1         PMSVTFQKYHLEHHRYQGVDGLDMDVPSLTEAKVVRNTVSKSIWVVLQLFFYALRPLFLK    180
WIMBERLEY_DN10949_c0_g1_i1      PMSVTFQKYHLEHHRYQGVDGLDMDVPSLTEAKVVRNTVSKSIWVVLQLFFYALRPLFLK    171
                                ************************************************************

SREEDHAR_DN8237_c0_g2_i1        PKPPGIWEFINFTIQLALDAAIVCLFGWKSFSYLILSTFVGGGMHPMAGHFISEHYVFNP    240
GUPTA_DN18238_c0_g1_i2          PKPPGIWEFINFTIQLALDAAIVCLFGWKSFSYLILSTFVGGGMHPMAGHFISEHYVFNP    231
PELAEZ_DN35633_c0_g1_i1         PKPPGIWEFINFTIQLALDAAIVCLFGWKSFSYLILSTFVGGGMHPMAGHFISEHYVFNP    240
WIMBERLEY_DN10949_c0_g1_i1      PKPPGIWEFINFTIQLALDAAIVCLFGWKSFSYLILSTFVGGGMHPMAGHFISEHYVFNP    231
                                ************************************************************

SREEDHAR_DN8237_c0_g2_i1        DQETYSYYGPLNLMTWSVGYHNEHHDFPRIPGSKLHKVREIAPQFYKDLDSYQSWSQVIY    300
GUPTA_DN18238_c0_g1_i2          DQETYSYYGPLNLMTWSVGYHNEHHDFPRIPGSKLHKVREIAPQFYKDLDSYQSWSQVIY    291
PELAEZ_DN35633_c0_g1_i1         DQETYSYYGPLNLMTWSVGYHNEHHDFPRIPGSKLHKVREIAPQFYKDLDSYQSWSQVIY    300
WIMBERLEY_DN10949_c0_g1_i1      DQETYSYYGPLNLMTWSVGYHNEHHDFPRIPGSKLHKVREIAPQFYKDLDSYQSWSQVIY    291
                                ************************************************************

SREEDHAR_DN8237_c0_g2_i1        MYIMDRTVGPFSRMKRSQSVLDKNKSE     327
GUPTA_DN18238_c0_g1_i2          MYIMDRTVGPFSRMKRSQSVLDKSKSE     318
PELAEZ_DN35633_c0_g1_i1         MYIMDRTVGPFSRMKRSQSVLDKSKSE     327
WIMBERLEY_DN10949_c0_g1_i1      MYIMDRTVGPFSRMKRSQSVLDKSKSE     318
                                ***********************.***
```

Alignment 3: These sequences bear high homology to A0A4D9AFL5_SALSN: “Omega-6 fatty acid desaturase (Delta-12 desaturase).”

```
CLUSTAL O(1.2.4) multiple sequence alignment


GUPTA_DN205_c2_g1_i3           MASRLAHSGFLILGPHKRPNEGNRIFPQSSTSSGSYLLKWESLPQRSIKQKQCLKSLQKR     60
PELAEZ_DN7729_c1_g3_i6         MASRLAHSGFLILGPHKRPNEGNRIFPQSSTSSGSYLLKWESLPQRSIKQKQCLKSLQKR     60
SREEDHAR_DN640_c2_g1_i1        MASRLAHSGFLILGPHKRPNEGNRIFPQSSTSSGSYLLKWESLPQRSIKQKQCLKSLQKR     60
WIMBERLEY_DN2561_c0_g3_i2      MASRLAHSGFLILGPHKRPNEGNRIFPQSSTSSGSYLLKWESLPQRSIKQKQCLKSLQKR     60
                               ************************************************************

GUPTA_DN205_c2_g1_i3           QVVKAVAVNVAPSPSADSAEYRQQLCHEYGFRQIGEPLPDNITLRDIIDTLPKKVFEIDD     120
PELAEZ_DN7729_c1_g3_i6         QVVKAVAVNVAPSPSADSAEYRQQLCHEYGFRQIGEPLPDNITLRDIIDTLPKKVFEIDD     120
SREEDHAR_DN640_c2_g1_i1        QVVKAVAVNVAPSPSADSAEYRQQLCHEYGFRQIGEPLPDNITLRDIIDTLPKKVFEIDD     120
WIMBERLEY_DN2561_c0_g3_i2      QVVKAVAVNVAPSPSADSAEYRQQLCHEYGFRQIGEPLPDNITLRDIIDTLPKKVFEIDD     120
                               ************************************************************

GUPTA_DN205_c2_g1_i3           AKALKSVLVSVTSYALGIFMITKSPWYLLPLAWAWTGTAITGFFVIGHDCAHKSFSKNKL     180
PELAEZ_DN7729_c1_g3_i6         AKALKSVLVSVTSYALGIFMITKSPWYLLPLAWAWTGTAITGFFVIGHDCAHKSFSKNKL     180
SREEDHAR_DN640_c2_g1_i1        AKALKSVLVSVTSYALGIFMITKSPWYLLPLAWAWTGTAITGFFVIGHDCAHKSFSKNKL     180
WIMBERLEY_DN2561_c0_g3_i2      AKALKSVLVSVTSYALGIFMITKSPWYLLPLAWAWTGTAITGFFVIGHDCAHKSFSKNKL     180
                               ************************************************************

GUPTA_DN205_c2_g1_i3           VEDIVGTLAFMPLIYPYEPWRFKHDRHHAKTNMLEEDTAWLPVNPEEFESSSLFRKAIIY     240
PELAEZ_DN7729_c1_g3_i6         VEDIVGTLAFMPLIYPYEPWRFKHDRHHAKTNMLEEDTAWLPVNPEEFESSSLFRKAIIY     240
SREEDHAR_DN640_c2_g1_i1        VEDIVGTLAFMPLIYPYEPWRFKHDRHHAKTNMLEEDTAWLPVNPEEFESSSLFRKAIIY     240
WIMBERLEY_DN2561_c0_g3_i2      VEDIVGTLAFMPLIYPYEPWRFKHDRHHAKTNMLEEDTAWLPVNPEEFESSSLFRKAIIY     240
                               ************************************************************

GUPTA_DN205_c2_g1_i3           AYGPLRPWMSIAHWLRMHFDVKKFRPNEVKRVKISLACVFGFMAIGWPLIILKTGVMGWI     300
PELAEZ_DN7729_c1_g3_i6         AYGPLRPWMSIAHWLRMHFDVKKFRPNEVKRVKISLACVFGFMAIGWPLIILKTGVMGWI     300
SREEDHAR_DN640_c2_g1_i1        AYGPLRPWMSIAHWLRMHFDVKKFRPNEVKRVKISLACVFGFMAIGWPLIILKTGVMGWI     300
WIMBERLEY_DN2561_c0_g3_i2      AYGPLRPWMSIAHWLRMHFDVKKFRPNEVKRVKISLACVFGFMAIGWPLIILKTGVMGWI     300
                               ************************************************************

GUPTA_DN205_c2_g1_i3           KFWLMPWLGYHFWMSTFTMVHHTAPHIPFKPSEEWNAAQAQLNGTVHCDYPSWIEILCHD     360
PELAEZ_DN7729_c1_g3_i6         KFWLMPWLGYHFWMSTFTMVHHTAPHIPFKPSEEWNAAQAQLNGTVHCDYPSWIEILCHD     360
SREEDHAR_DN640_c2_g1_i1        KFWLMPWLGYHFWMSTFTMVHHTAPHIPFKPSEEWNAAQAQLNGTVHCDYPSWIEILCHD     360
WIMBERLEY_DN2561_c0_g3_i2      KFWLMPWLGYHFWMSTFTMVHHTAPHIPFKPSEEWNAAQAQLNGTVHCDYPSWIEILCHD     360
                               ************************************************************

GUPTA_DN205_c2_g1_i3           INVHIPHHISPRIPSYNLRVAHQSLQENWGKYLNEASWNWRLMKTILTVCHVYSKEKNYI     420
PELAEZ_DN7729_c1_g3_i6         INVHIPHHISPRIPSYNLRAAHQSLQENWGKYLNEASWNWRLMKTILTVCHVYSKEKNYI     420
SREEDHAR_DN640_c2_g1_i1        INVHIPHHISPRIPSYNLRAAHQSLQENWGKYLNEASWNWRLMKTILTVCHVYSKEKNYI     420
WIMBERLEY_DN2561_c0_g3_i2      INVHIPHHISPRIPSYNLRAAHQSLQENWGKYLNEASWNWRLMKTILTVCHVYSKEKNYI     420
                               *******************.****************************************

GUPTA_DN205_c2_g1_i3           PFDELAPDDSQPITFLKQVMPDYA 444
PELAEZ_DN7729_c1_g3_i6         PFDELAPDDSQPITFLKQVMPDYA 444
SREEDHAR_DN640_c2_g1_i1        PFDELAPDDSQPITFLKQVMPDYA 444
WIMBERLEY_DN2561_c0_g3_i2      PFDELAPDDSQPITFLKQVMPDYA 444
                               ************************
```

Alignment 4: These sequences bear high homology to A0A4D8Z2A5_SALSN: “Delta8-fatty-acid desaturase.”

```
CLUSTAL O(1.2.4) multiple sequence alignment


GUPTA_DN1918_c0_g1_i3           MAETKKYISAEELATHNKPGDLWISIQGKIYDVSDWIKSHPGGDLPLLNLAGRDATDAFV    60
PELAEZ_DN3148_c0_g3_i1          MAETKKYISAEELASHNKPGDLWISIQGKIYDVSDWIKSHPGGDLPLLNLAGRDATDAFV    60
SREEDHAR_DN1698_c0_g1_i2        MAETKKYISAEELASHNKPGDLWISIQGKIYDVSDWIKSHPGGDLPLLNLAGRDATDAFV    60
WIMBERLEY_DN1165_c0_g2_i3       MAETKKYISAEELASHNKPGDLWISIQGKIYDVSDWIKSHPGGDLPLLNLAGRDATDAFV    60
                                **************:*********************************************

GUPTA_DN1918_c0_g1_i3           AYHPAHAWSHLGPFHNHLHLQNYVVSDVSKDYRKLVHEFTKLGLFDKKGHNVLFSLTAIA    120
PELAEZ_DN3148_c0_g3_i1          AYHPAHAWSHLAPFHNHLHLQNYVVSEVSKDYRKLVHEFTKLGLFDKKGHNVLFSLTAIA    120
SREEDHAR_DN1698_c0_g1_i2        AYHPAHAWSHLAPFHNHLHLQNYVVSEVSKDYRKLVHEFTKLGLFDKKGHNVLFSLTAIA    120
WIMBERLEY_DN1165_c0_g2_i3       AYHPAHAWSHLAPFHNHLHLQNYVVSEVSKDYRKLVHEFTKLGLFDKKGHNVLFSLTAIA    120
                                ***********.**************:*********************************

GUPTA_DN1918_c0_g1_i3           MLFSLCLYGVVFCHGALLHALCGGAMGFLWIQSGWLGHDSGHYQIMLNRSSNRFIQVLSG    180
PELAEZ_DN3148_c0_g3_i1          MLFSLCLYGVVFCHGALLHALCGGAMGFLWIQSGWLGHDSGHYQIMLNRSSNRFIQVLSG    180
SREEDHAR_DN1698_c0_g1_i2        MLFSLCLYGVVFCHGALLHALCGGAMGFLWIQSGWLGHDSGHYQIMLNRSSNRFIQVLSG    180
WIMBERLEY_DN1165_c0_g2_i3       MLFSLCLYGVVFCHGALLHALCGGAMGFLWIQSGWLGHDSGHYQIMLNRSSNRFIQVLSG    180
                                ************************************************************

GUPTA_DN1918_c0_g1_i3           NCLAGISIAWWKRNHNAHHIACNSLDHDPDLQHMPFFAVSPKFFNSITSLYYNRKLEFDK    240
PELAEZ_DN3148_c0_g3_i1          NCLAGISIAWWKRNHNAHHIACNSLDHDPDLQHMPFFAVSPKFFNSITSLYYNRKLEFDK    240
SREEDHAR_DN1698_c0_g1_i2        NCLAGISIAWWKRNHNAHHIACNSLDHDPDLQHMPFFAVSPKFFNSITSLYYNRKLEFDK    240
WIMBERLEY_DN1165_c0_g2_i3       NCLAGISIAWWKRNHNAHHIACNSLDHDPDLQHMPFFAVSPKFFNSITSLYYNRKLEFDK    240
                                ************************************************************

GUPTA_DN1918_c0_g1_i3           FARFLISKQHFTYYPVMSLARLNLFAQSFFLLSSNKDVPNRVQEILGLLTFWIWYPLLVS    300
PELAEZ_DN3148_c0_g3_i1          FARFLISKQHFTYYPVMSLARLNLFAQSFFLLSSNKDVPNRVQEILGLLTFWIWYPLLVS    300
SREEDHAR_DN1698_c0_g1_i2        FARFLISKQHFTYYPVMSLARLNLFAQSFFLLSSNKDVPNRVQEILGLLTFWIWYPLLVS    300
WIMBERLEY_DN1165_c0_g2_i3       FARFLISKQHFTYYPVMSLARLNLFAQSFFLLSSNKDVPNRVQEILGLLTFWIWYPLLVS    300
                                ************************************************************

GUPTA_DN1918_c0_g1_i3           FLPNWKEKLMFVACSFVVTSIQHVQFTLNHFSANVYVGLPKGNDWFEKQTYGTLNISCPP    360
PELAEZ_DN3148_c0_g3_i1          FLPNWKEKLMFVACSFVVTSIQHVQFTLNHFSANVYVGLPKGNDWFEKQTYGTLNISCPP    360
SREEDHAR_DN1698_c0_g1_i2        FLPNWKEKLMFVACSFVVTSIQHVQFTLNHFSANVYVGLPKGNDWFEKQTYGTLNISCPP    360
WIMBERLEY_DN1165_c0_g2_i3       FLPNWKEKLMFVACSFVVTSIQHVQFTLNHFSANVYVGLPKGNDWFEKQTYGTLNISCPP    360
                                ************************************************************

GUPTA_DN1918_c0_g1_i3           WMDWFHGGLQFQIEHHLFPRLPRSQLRGVAPFVKELCKKHGLPYNCATFWGANVMTLRTL    420
PELAEZ_DN3148_c0_g3_i1          WMDWFHGGLQFQIEHHLFPRLPRSQLRGVAPFVKELCKKHGLPYNCATFWGANVMTLRTL    420
SREEDHAR_DN1698_c0_g1_i2        WMDWFHGGLQFQIEHHLFPRLPRSQLRGVAPFVKELCKKHGLPYNCATFWGANVMTLRTL    420
WIMBERLEY_DN1165_c0_g2_i3       WMDWFHGGLQFQIEHHLFPRLPRSQLRGVAPFVKELCKKHGLPYNCATFWGANVMTLRTL    420
                                ************************************************************

GUPTA_DN1918_c0_g1_i3           RAAAMQARDYSKPAPRNLVWEAVNSIG     447
PELAEZ_DN3148_c0_g3_i1          RAAAMQARDYSKPAPRNLVWEAVNSIG     447
SREEDHAR_DN1698_c0_g1_i2        RAAAMQARDYSKPAPRNLVWEAVNSIG     447
WIMBERLEY_DN1165_c0_g2_i3       RAAAMQARDYSKPAPRNLVWEAVNSIG     447
                                ***************************
```

Alignment 5: These sequences bear high homology to A0A4D9BNI1_SALSN: “Stearoyl-CoA desaturase (Delta-9 desaturase)”

```
CLUSTAL O(1.2.4) multiple sequence alignment


GUPTA_DN171_c6_g1_i3            MALLAPPPSNLKPFQFPSLHRFNPLKITHKHQNSIHFASNARFPQNSLKDSIFFRRKDAK    60
PELAEZ_DN21775_c0_g1_i1         MALLAPPPSNLKPFQFPSLHRFNPLKITHKHQNSIHFASNARFPQNSLKDSIFFRRKDAK    60
SREEDHAR_DN7287_c0_g1_i1        MALLAPPPSNLKPFQFPSLHRFNPLKITHKHQNSIHFASNARFPQNSLKDSIFFRRKDAK    60
WIMBERLEY_DN6716_c0_g1_i38      ------------------------------------------------------------    0


GUPTA_DN171_c6_g1_i3            ITRVSPIVRAASIPLSGDEKDSNFGRILFSDVVVKRKRNVFWSRKWNSLDIGIVSVVAAM    120
PELAEZ_DN21775_c0_g1_i1         ITRVSPIVRAASIPLSGDEKDSNFGRILFSDVVVKRKRNVFWSRKWNSLDIGIVSVVAAM    120
SREEDHAR_DN7287_c0_g1_i1        ITRVSPIVRAASIPLSGDEKDSNFGRILFSDVVVKRKRNVFWSRKWNSLDIGIVSVVAAM    120
WIMBERLEY_DN6716_c0_g1_i38      -----------------------------------------------------------M    1
                                                                                           *

GUPTA_DN171_c6_g1_i3            HLLCVLAPSTFNWGALGVAVALYVITGLLGITLSFHRNLSHRSFKLPKWLEYFFAYCGSL    180
PELAEZ_DN21775_c0_g1_i1         HLLCVLAPSTFNWGALGVAVALYVITGLLGITLSFHRNLSHRSFKLPKWLEYFFAYCGSL    180
SREEDHAR_DN7287_c0_g1_i1        HLLCVLAPSTFNWGALGVAVALYVITGLLGITLSFHRNLSHRSFKLPKWLEYFFAYCGSL    180
WIMBERLEY_DN6716_c0_g1_i38      HLLCVLAPSTFNWGALGVAVALYVITGLLGITLSFHRNLSHRSFKLPKWLEYFFAYCGSL    61
                                ************************************************************

GUPTA_DN171_c6_g1_i3            ALQGNPIEWVSTHRFHHQFCDSERDPHSPIEGFWFSHISWFFDSENITQRCGEPTNVGDL    240
PELAEZ_DN21775_c0_g1_i1         ALQGNPIEWVSTHRFHHQFCDSERDPHSPIEGFWFSHISWFFDSENITQRCGEPTNVGDL    240
SREEDHAR_DN7287_c0_g1_i1        ALQGNPIEWVSTHRFHHQFCDSERDPHSPIEGFWFSHISWFFDSENITQRCGEPTNVGDL    240
WIMBERLEY_DN6716_c0_g1_i38      ALQGNPIEWVSTHRFHHQFCDSERDPHSPIEGFWFSHISWFFDSENITQRCGEPTNVGDL    121
                                ************************************************************

GUPTA_DN171_c6_g1_i3            EKQPFYKFLQNTYILHQVALGALLYAMGGLPFIVWGIGVRTVWVYHITWLVNSACHVWGK    300
PELAEZ_DN21775_c0_g1_i1         EKQPFYKFLQNTYILHQVALGALLYAMGGLPFIVWGIGVRTVWVYHITWLVNSACHVWGK    300
SREEDHAR_DN7287_c0_g1_i1        EKQPFYKFLQNTYILHQVALGALLYAMGGLPFIVWGIGVRTVWVYHITWLVNSACHVWGK    300
WIMBERLEY_DN6716_c0_g1_i38      EKQPFYKFLQNTYILHQVALGALLYAMGGLPFIVWGIGVRTVWVYHITWLVNSACHVWGK    181
                                ************************************************************

GUPTA_DN171_c6_g1_i3            QAWNTGDLSRNNWWVAVLAFGEGWHNNHHAFEYSARHGLEWWQIDMTWYAIRALEALGLA    360
PELAEZ_DN21775_c0_g1_i1         QAWNTGDLSRNNWWVAVLAFGEGWHNNHHAFEYSARHGLEWWQIDMTWYAIRALEALGLA    360
SREEDHAR_DN7287_c0_g1_i1        QAWNTGDLSRNNWWVAVLAFGEGWHNNHHAFEYSARHGLEWWQIDMTWYAIRALEALGLA    360
WIMBERLEY_DN6716_c0_g1_i38      QAWNTGDLSRNNWWVAVLAFGEGWHNNHHAFEYSARHGLEWWQIDMTWYAIRVLEALGLA    241
                                ****************************************************.*******

GUPTA_DN171_c6_g1_i3            TDVKLPTPAQKQKMKALN      378
PELAEZ_DN21775_c0_g1_i1         TDVKLPTPAQKQKMKALN      378
SREEDHAR_DN7287_c0_g1_i1        TDVKLPTPAQKQKMKALN      378
WIMBERLEY_DN6716_c0_g1_i38      ------------------      241
```

Alignment 6 (next page): This long alignment bears homology to A0A1Z1EC52 _9LAMI: "Fatty acid desaturase 8." This is a rare example of a sequence from *S. hispanica* that is already present in the UniProt Knowledgebase. Note the substantial section of the 5' end of the sequence that is absent in the transcript assembled from Peláez Celaya. The large gap in the penultimate row of the alignment has arisen from several amino acids that are inserted into one of the isoforms from the Wimberley sequence. The isoform may be genuine or the insertion may have resulted from an assembly error.

```
CLUSTAL O(1.2.4) multiple sequence alignment


WIMBERLEY_DN3016_c0_g2_i1       -MEREHKLLPLAYFREVEPFINLSTLFHPNQLALSFHIQTTFPQLPQVHNFLHRERAPLK    59
GUPTA_DN76_c0_g4_i1             ------------------------------------------------------------    0
PELAEZ_DN30117_c0_g1_i1         ------------------------------------------------------------    0
SREEDHAR_DN6151_c0_g1_i1        ------------------------------------------------------------    0
WIMBERLEY_DN3016_c0_g2_i4       ------------------------------------------------------------    0
WIMBERLEY_DN3016_c0_g2_i4b      YMEREHKLLPLAYFREVEPFINLSTLFHPNQLALSFHIQTTFPQLPQVHNFLHRERAPLK    60


WIMBERLEY_DN3016_c0_g2_i1       KVKSLLDCLSLSLMASFVISGCGLKPLPRIYPKPRSVQNSFSTSNLRISRPNQFSSSSIG    119
GUPTA_DN76_c0_g4_i1             -------------MASFVISGCGLKPLPRIYPKPRSVQNSFSTSNLRISRPNQFSSSSIG    47
PELAEZ_DN30117_c0_g1_i1         ------------------------------------------------------------    0
SREEDHAR_DN6151_c0_g1_i1        -------------MASFVISGCGLKPLPRIYPKPRSVQNSFSTSNLRISRPNQFSSSSIG    47
WIMBERLEY_DN3016_c0_g2_i4       -------------MASFVISGCGLKPLPRIYPKPRSVQNSFSTSNLRISRPNQFSSSSIG    47
WIMBERLEY_DN3016_c0_g2_i4b      KVKSLLDCLSLSLMASFVISGCGLKPLPRIYPKPRSVQNSFSTSNLRISRPNQFSSSSIG    120


WIMBERLEY_DN3016_c0_g2_i1       INQKRNWGLGVSAPLRIQPLEEENEEFDPAAPPPFKLSDIKAAIPKHCWVKDPWRSVGYV    179
GUPTA_DN76_c0_g4_i1             INQKRNWGLGVSAPLRIQPLEEENEEFDPAAPPPFKLSDIKAAIPKHCWVKDPWRSVGYV    107
PELAEZ_DN30117_c0_g1_i1         ------------------------------------------------------------    0
SREEDHAR_DN6151_c0_g1_i1        INQKRNWGLGVSAPLRIQPLEEENEEFDPAAPPPFKLSDIKAAIPKHCWVKDPWRSVGYV    107
WIMBERLEY_DN3016_c0_g2_i4       INQKRNWGLGVSAPLRIQPLEEENEEFDPAAPPPFKLSDIKAAIPKHCWVKDPWRSVGYV    107
WIMBERLEY_DN3016_c0_g2_i4b      INQKRNWGLGVSAPLRIQPLEEENEEFDPAAPPPFKLSDIKAAIPKHCWVKDPWRSVGYV    180


WIMBERLEY_DN3016_c0_g2_i1       VRDWVAVLGMAAAAAYFNSWIVWPLYWFAQSTMFWALFVLGHDCGHGSFSNNPKLNSVFG    239
GUPTA_DN76_c0_g4_i1             VRDWVAVLGMAAAAAYFNSWIVWPLYWFAQSTMFWALFVLGHDCGHGSFSNNPKLNSVFG    167
PELAEZ_DN30117_c0_g1_i1         ------------------------------------------------------------    0
SREEDHAR_DN6151_c0_g1_i1        VRDWVAVLGMAAAAAYFNSWIVWPLYWFAQSTMFWALFVLGHDCGHGSFSNNPKLNSVFG    167
WIMBERLEY_DN3016_c0_g2_i4       VRDWVAVLGMAAAAAYFNSWIVWPLYWFAQSTMFWALFVLGHDCGHGSFSNNPKLNSVFG    167
WIMBERLEY_DN3016_c0_g2_i4b      VRDWVAVLGMAAAAAYFNSWIVWPLYWFAQSTMFWALFVLGHDCGHGSFSNNPKLNSVFG    240


WIMBERLEY_DN3016_c0_g2_i1       HFLHSSILVPYHGWRISHRTHHQNHGHVENDESWHPMPEKIYNSLDSMAKKLRFTLPFPM    299
GUPTA_DN76_c0_g4_i1             HFLHSSILVPYHGWRISHRTHHQNHGHVENDESWHPMPEKIYNSLDSMAKKLRFTLPFPM    227
PELAEZ_DN30117_c0_g1_i1         ------------------------------------MPEKIYNSLDSMAKKLRFTLPFPM    24
SREEDHAR_DN6151_c0_g1_i1        HFLHSSILVPYHGWRISHRTHHQNHGHVENDESWHPMPEKIYNSLDSMAKKLRFTLPFPM    227
WIMBERLEY_DN3016_c0_g2_i4       HFLHSSILVPYHGWRISHRTHHQNHGHVENDESWHPMPEKIYNSLDSMAKKLRFTLPFPM    227
WIMBERLEY_DN3016_c0_g2_i4b      HFLHSSILVPYHGWRISHRTHHQNHGHVENDESWHPMPEKIYNSLDSMAKKLRFTLPFPM    300
                                                                    ************************

WIMBERLEY_DN3016_c0_g2_i1       LAYPIYLWTRSPGKKGSHYHPDSDLFVPAERKDVITSTVCWTAMAALLVGLSFVMGPIQL    359
GUPTA_DN76_c0_g4_i1             LAYPIYLWTRSPGKKGSHYHPDSDLFVPAERKDVITSTVCWTAMAALLVGLSFVMGPIQL    287
PELAEZ_DN30117_c0_g1_i1         LAYPIYLWTRSPGKKGSHYHPDSDLFVPAERKDVITSTVCWTAMAALLVGLSFVMGPIQL    84
SREEDHAR_DN6151_c0_g1_i1        LAYPIYLWTRSPGKKGSHYHPDSDLFVPAERKDVITSTVCWTAMAALLVGLSFVMGPIQL    287
WIMBERLEY_DN3016_c0_g2_i4       LAYPIYLWTRSPGKKGSHYHPDSDLFVPAERKDVITSTVCWTAMAALLVGLSFVMGPIQL    287
WIMBERLEY_DN3016_c0_g2_i4b      LAYPIYLWTRSPGKKGSHYHPDSDLFVPAERKDVITSTVCWTAMAALLVGLSFVMGPIQL    360
                                ************************************************************

WIMBERLEY_DN3016_c0_g2_i1       LKLYGIPYLGFVAWLDTVTYLHHHGHEDKLPWYRGKEWSYLRGGLTTLDRDYGLINNIHH    419
GUPTA_DN76_c0_g4_i1             LKLYGIPYLGFVAWLDTVTYLHHHGHEDKLPWYRGKEWSYLRGGLTTLDRDYGLINNIHH    347
PELAEZ_DN30117_c0_g1_i1         LKLYGIPYLGFVAWLDTVTYLHHHGHEDKLPWYRGKEWSYLRGGLTTLDRDYGLINNIHH    144
SREEDHAR_DN6151_c0_g1_i1        LKLYGIPYLGFVAWLDTVTYLHHHGHEDKLPWYRGKEWSYLRGGLTTLDRDYGLINNIHH    347
WIMBERLEY_DN3016_c0_g2_i4       LKLYGIPYLGFVAWLDTVTYLHHHGHEDKLPWYRGKEWSYLRGGLTTLDRDYGLINNIHH    347
WIMBERLEY_DN3016_c0_g2_i4b      LKLYGIPYLGFVAWLDTVTYLHHHGHEDKLPWYRGKEWSYLRGGLTTLDRDYGLINNIHH    420
                                ************************************************************

WIMBERLEY_DN3016_c0_g2_i1       DIGTHVIHHLFPQIPHYNLIEAVSFFSIFKGITLPQTVELVLHVFDS-AWPILAMQTEAA    478
GUPTA_DN76_c0_g4_i1             DIGTHVIHHLFPQIPHYNLIEA----------------------------------TEAA    373
PELAEZ_DN30117_c0_g1_i1         DIGTHVIHHLFPQIPHYNLIEA----------------------------------TEAA    170
SREEDHAR_DN6151_c0_g1_i1        DIGTHVIHHLFPQIPHYNLIEA----------------------------------TEAA    373
WIMBERLEY_DN3016_c0_g2_i4       DIGTHVIHHLFPQIPHYNLIEA----------------------------------TEAA    373
WIMBERLEY_DN3016_c0_g2_i4b      DIGTHVIHHLFPQIPHYNLIEA----------------------------------TEAA    446
                                **********************                         *        ****

WIMBERLEY_DN3016_c0_g2_i1       KGVLGKYYREPKKSGPLPLHLLGDLVRSLKKDHYVSDTGDVVYYQTDPQLNGGQKS        534
GUPTA_DN76_c0_g4_i1             KGVLGKYYREPKKSGPLPLHLLGDLVRSLKKDHYVSDTGDVVYYQTDPQLNGGQKS        429
PELAEZ_DN30117_c0_g1_i1         KGVLGKYYREPKKSGPLPLHLLGDLVRSLKKDHYVSDTGDVVYYQTDPQLNGGQKS        226
SREEDHAR_DN6151_c0_g1_i1        KGVLGKYYREPKKSGPLPLHLLGDLVRSLKKDHYVSDTGDVVYYQTDPQLNGGQKS        429
WIMBERLEY_DN3016_c0_g2_i4       KGVLGKYYREPKKSGPLPLHLLGDLVRSLKKDHYVSDTGDVVYYQTDPQLNGGQKS        429
WIMBERLEY_DN3016_c0_g2_i4b      KGVLGKYYREPKKSGPLPLHLLGDLVRSLKKDHYVSDTGDVVYYQTDPQLNGGQKS        502
                                ********************************************************
```

Alignment 7: This alignment bears high homology to A0A4D9B0B7_SALSN: “Delta8-fatty-acid desaturase.”

```
CLUSTAL O(1.2.4) multiple sequence alignment


WIMBERLEY_DN1165_c0_g1_i5      MAAAAAAADDKKYITSEELSKHNKSGDLWLSIQGKVYDVTDWAKQHPGGEVPLLNLGGQD    60
GUPTA_DN2093_c1_g1_i8          MAAAAAAADDKKYITSEELSKHNKSGDLWLSIQGKVYDVTDWAKQHPGGEVPLLNLGGQD    60
GUPTA_DN2093_c1_g1_i6          MAAAAAAADDKKYITSEELSKHNKSGDLWLSIQGKVYDVTDWAKQHPGGEVPLLNLGGQD    60
GUPTA_DN2093_c1_g1_i7          MAAAAAAADDKKYITSEELSKHNKSGDLWLSIQGKVYDVTDWAKQHPGGEVPLLNLGGQD    60
PELAEZ_DN3148_c0_g1_i8         MAAAAAAADDKKYITSEELSKHNKSGDLWLSIQGKVYDVTDWAKQHPGGEVPLLNLGGQD    60
SREEDHAR_DN6761_c0_g3_i5       MAAAAAAADDKKYITSEELSKHNKSGDLWLSIQGKVYDVTDWAKQHPGGEVPLLNLGGQD    60
                               ************************************************************

WIMBERLEY_DN1165_c0_g1_i5      VTDAFIAFHPGSAWKHLDNFFTGYHLRDFHVSDMSRDYRNLATQFTRSGMFEKKGHGVIY    120
GUPTA_DN2093_c1_g1_i8          VTDAFIAFHPGSAWKHLDSLFTGYHLRDFHVSDMSRDYRNLATQFTRSGMFEKKGHGVIY    120
GUPTA_DN2093_c1_g1_i6          VTDAFIAFHPGSAWKHLDSLFTGYHLRDFHVSDMSRDYRNLATQFTRSGMFEKKGHGVIY    120
GUPTA_DN2093_c1_g1_i7          VTDAFIAFHPGSAWKHLDSLFTGYHLRDFHVSDMSRDYRNLATQFTRSGMFEKKGHGVIY    120
PELAEZ_DN3148_c0_g1_i8         VTDAFIAFHPGSAWKHLDSLFTGYHLRDFHVSDMSRDYRNLATQFTRSGMFEKKGHGVIY    120
SREEDHAR_DN6761_c0_g3_i5       VTDAFIAFHPGSAWKHLDSLFTGYHLRDFHVSDMSRDYRNLATQFTRSGMFEKKGHGVIY    120
                               ******************.:****************************************

WIMBERLEY_DN1165_c0_g1_i5      SLCFVSFLLAACFYGVLGCDGFLTHILSGGLLGLIWMQVAYLGHDSGHYNIMISPRFNKL    180
GUPTA_DN2093_c1_g1_i8          SLCFVSFLLAACFYGVLRCDGFLTHILSGGLLGLIWMQVAYLGHDSGHYNIMISPRFNKL    180
GUPTA_DN2093_c1_g1_i6          SLCFVSFLLAACFYGVLRCDGFLTHILSGGLLGLIWMQVAYLGHDSGHYNIMISPRFNKL    180
GUPTA_DN2093_c1_g1_i7          SLCFVSFLLAACFYGVLRCDGFLTHILSGGLLGLIWMQVAYLGHDSGHYNIMISPRFNKL    180
PELAEZ_DN3148_c0_g1_i8         SLCFISVLLAACFYGVLRCDGFLTHILSGGLLGLIWMQVAYLGHDSGHYNIMISPRFNKL    180
SREEDHAR_DN6761_c0_g3_i5       SLCFISVLLAACFYGVLRCDGFLTHILSGGLLGLIWMQVAYLGHDSGHYNIMISPRFNKL    180
                               ****:*.********** ******************************************

WIMBERLEY_DN1165_c0_g1_i5      AQILTGNCLTGISIAWWKWTHNAHHIACNSLDYDPDLQHLPMLAVSTRFFTNLTSKFYNR    240
GUPTA_DN2093_c1_g1_i8          AQILTGNCLTGISIAWWKWTHNAHHIACNSLDYDPDLQHLPMLAVSTRFFTNLTSKFYNR    240
GUPTA_DN2093_c1_g1_i6          AQILTGNCLTGISIAWWKWTHNAHHIACNSLDYDPDLQHLPMLAVSTRFFTNLTSKFYNR    240
GUPTA_DN2093_c1_g1_i7          AQILTGNCLTGISIAWWKWTHNAHHIACNSLDYDPDLQHLPMLAVSTRFFTNLTSKFYNR    240
PELAEZ_DN3148_c0_g1_i8         AQILTGNCLTGISIAWWKWTHNAHHIACNSLDYDPDLQHLPMLAVSTRFFTNLTSKFYNR    240
SREEDHAR_DN6761_c0_g3_i5       AQILTGNCLTGISIAWWKWTHNAHHIACNSLDYDPDLQHLPMLAVSTRFFTNLTSKFYNR    240
                               ************************************************************

WIMBERLEY_DN1165_c0_g1_i5      KLEFDPIARFFISYQHLTYYPVMCVARVNLYLQTFLLLLSNRRVPDRALNILGIMIFWTW    300
GUPTA_DN2093_c1_g1_i8          KLEFDPIARFFISYQHLTYYPVMCVARVNLYLQTFLLLLSNRRVPDRALNILGIMIFWTW    300
GUPTA_DN2093_c1_g1_i6          KLEFDPIARFFISYQHLTYYPVMCVARVNLYLQTFLLLLSNRRVPDRALNILGIMIFWTW    300
GUPTA_DN2093_c1_g1_i7          KLEFDPIARFFISYQHLTYYPVMCVARVNLYLQTFLLLLSNRRVPDRALNILGIMIFWTW    300
PELAEZ_DN3148_c0_g1_i8         KLEFDPIARFFISYQHLTYYPVMCVARVNLYLQTFLLLLSNRRVPDRALNILGIMIFWTW    300
SREEDHAR_DN6761_c0_g3_i5       KLEFDPIARFFISYQHLTYYPVMCVARVNLYLQTFLLLLSNRRVPDRALNILGIMIFWTW    300
                               ************************************************************

WIMBERLEY_DN1165_c0_g1_i5      FPLLVSCLPNWTERVLFVLASFCVCAIQHIQFTLNHFAADVYVGPPKGNNWFEKQTAGTI    360
GUPTA_DN2093_c1_g1_i8          FPLLVSCLPNWTERVLFVLASFCVCSIQHIQFTLNHFAADVYVGPPKGNNWFEKQTAGTI    360
GUPTA_DN2093_c1_g1_i6          FPLLVSCLPNWTERVLFVLASFCVCSIQHIQFTLNHFAADVYVGPPKGNNWFEKQTAGTI    360
GUPTA_DN2093_c1_g1_i7          FPLLVSCLPNWTERVLFVLASFCVCSIQHIQFTLNHFAADVYVGPPKGNNWFEKQTAGTI    360
PELAEZ_DN3148_c0_g1_i8         FPLLVSCLPNWTERVLFVLASFCVCAIQHIQFTLNHFAADVYVGPPKGNNWFEKQTAGTI    360
SREEDHAR_DN6761_c0_g3_i5       FPLLVSCLPNWTERVLFVLASFCVCSIQHIQFTLNHFAADVYVGPPKGNNWFEKQTAGTI    360
                               *************************:**********************************

WIMBERLEY_DN1165_c0_g1_i5      DIDCPSWMDWFFGGLQFQLEHHLFPRLPRCHLRKVSPIIRDLCKKHNLPYRSLTFVEANK    420
GUPTA_DN2093_c1_g1_i8          DIDCPSWMDWFFGGLQFQLEHHLFPRLPRCHLRKVSPIIRDLCKKHNLPYRSLTFVEANK    420
GUPTA_DN2093_c1_g1_i6          DIDCPSWMDWFFGGLQFQLEHHLFPRLPRCHLRKVSPIIRDLCKKHNLPYRSLTFVEANK    420
GUPTA_DN2093_c1_g1_i7          DIDCPSWMDWFFGGLQFQLEHHLFPRLPRCHLRKVSPIIRDLCKKHNLPYRSLTFVEANK    420
PELAEZ_DN3148_c0_g1_i8         DIDCPSWMDWFFGGLQFQLEHHLFPRLPRCHLRKVSPIIRDLCKKHNLPYRSLTFVEANK    420
SREEDHAR_DN6761_c0_g3_i5       DIDCPSWMDWFFGGLQFQLEHHLFPRLPRCHLRKVSPIIRDLCKKHNLPYRSLTFVEANK    420
                               ************************************************************

WIMBERLEY_DN1165_c0_g1_i5      WTLRTLRTAAVEARDFSMAPRNLLWEAVNTHG 452
GUPTA_DN2093_c1_g1_i8          WTLRTLRTAAVEARDFSMAPRNLLWEAVNTHG 452
GUPTA_DN2093_c1_g1_i6          WTLRTLRTAAVEARDFSMAPRNLLWEAVNTHG 452
GUPTA_DN2093_c1_g1_i7          WTLRTLRTAAVEARDFSMAPRNLLWEAVNTHG 452
PELAEZ_DN3148_c0_g1_i8         WTLRTLRTAAVEARDFSMAPRNLLWEAVNTHG 452
SREEDHAR_DN6761_c0_g3_i5       WTLRTLRTAAVEARDFSMAPRNLLWEAVNTHG 452
                               ********************************
```

Alignment 8: Each of the sequences in this alignment represents exactly the same "gene" clusters from Trinity as in Alignment 7, but in every case Alignment 8 represents a different isoform than represented the genes in Alignment 7. The smaller height of this image may create the impression that Alignment 8's isoforms are shorter than those of Alignment 7, but it is an illusion since Alignment 7 includes three isoforms from GUPTA_DN2093 while Alignment 8 includes only one. It is unsurprising that it also bears high homology to A0A4D9B0B7_SALSN: "Delta8-fatty-acid desaturase."

```
CLUSTAL O(1.2.4) multiple sequence alignment


WIMBERLEY_DN1165_c0_g1_i4      MAAAAAAADDKKYITSEELSKHNKSGDLWLSIQGKVYDVTDWAKQHPGGEVPLLNLGGQD     60
GUPTA_DN2093_c1_g1_i9          MAAAAAAADDKKYITSEELSKHNKSGDLWLSIQGKVYDVTDWAKQHPGGEVPLLNLGGQD     60
PELAEZ_DN3148_c0_g1_i11        MAAAAAAADDKKYITSEELSKHNKSGDLWLSIQGKVYDVTDWAKQHPGGEVPLLNLGGQD     60
SREEDHAR_DN6761_c0_g3_i4       MAAAAAAADDKKYITSEELSKHNKSGDLWLSIQGKVYDVTDWAKQHPGGEVPLLNLGGQD     60
                               ************************************************************

WIMBERLEY_DN1165_c0_g1_i4      VTDAFIAFHPGSAWKHLDNFFTGYHLRDFHVSDMSRDYRNLATQFTRSGMFEKKGHGVIY     120
GUPTA_DN2093_c1_g1_i9          VTDAFIAFHPGSAWKHLDSLFTGYHLRDFHVSDMSRDYRNLATQFTRSGMFEKKGHGVIY     120
PELAEZ_DN3148_c0_g1_i11        VTDAFIAFHPGSAWKHLDSLFTGYHLRDFHVSDMSRDYRNLATQFTRSGMFEKKGHGVIY     120
SREEDHAR_DN6761_c0_g3_i4       VTDAFIAFHPGSAWKHLDSLFTGYHLRDFHVSDMSRDYRNLATQFTRSGMFEKKGHGVIY     120
                               ******************.:****************************************

WIMBERLEY_DN1165_c0_g1_i4      SLCFVSFLLAACFYGVLGCDGFLTHILSGGLLGLIWMQVAYLGHDSGHYNIMISPRFNKL     180
GUPTA_DN2093_c1_g1_i9          SLCFVSFLLAACFYGVLRCDGFLTHILSGGLLGLIWMQVAYLGHDSGHYNIMISPRFNKL     180
PELAEZ_DN3148_c0_g1_i11        SLCFISVLLAACFYGVLRCDGFLTHILSGGLLGLIWMQVAYLGHDSGHYNIMISPRFNKL     180
SREEDHAR_DN6761_c0_g3_i4       SLCFISVLLAACFYGVLRCDGFLTHILSGGLLGLIWMQVAYLGHDSGHYNIMISPRFNKL     180
                               ****:*.********** ******************************************

WIMBERLEY_DN1165_c0_g1_i4      AQILTGNCLTGISIAWWKWTHNAHHIACNSLDYDPDLQHLPMLAVSTRFFTNLTSKFYNR     240
GUPTA_DN2093_c1_g1_i9          AQILTGNCLTGISIAWWKWTHNAHHIACNSLDYDPDLQHLPMLAVSTRFFTNLTSKFYNR     240
PELAEZ_DN3148_c0_g1_i11        AQILTGNCLTGISIAWWKWTHNAHHIACNSLDYDPDLQHLPMLAVSTRFFTNLTSKFYNR     240
SREEDHAR_DN6761_c0_g3_i4       AQILTGNCLTGISIAWWKWTHNAHHIACNSLDYDPDLQHLPMLAVSTRFFTNLTSKFYNR     240
                               ************************************************************

WIMBERLEY_DN1165_c0_g1_i4      KLEFDPIARFFISYQHLTYYPVMCVARVNLYLQTFLLLLSSRRVPDRGLNILGIMVFWTW     300
GUPTA_DN2093_c1_g1_i9          KLEFDPIARFFISYQHLTYYPVMCVARVNLYLQTFLLLLSSRRVPDRGLNILGIMVFWTW     300
PELAEZ_DN3148_c0_g1_i11        KLEFDPIARFFISYQHLTYYPVMCVARVNLYLQTFLLLLSSRRVPDRGLNILGIMVFWTW     300
SREEDHAR_DN6761_c0_g3_i4       KLEFDPIARFFISYQHLTYYPVMCVARVNLYLQTFLLLLSSRRVPDRGLNILGIMVFWTW     300
                               ************************************************************

WIMBERLEY_DN1165_c0_g1_i4      FPLLVSFLPNWTERVLFVLASFCVCAIQHIQFTLNHFAADVYVGPPKGNNWFEKQTAGTI     360
GUPTA_DN2093_c1_g1_i9          FPLLVSFLPNWTERVLFVLASFCVCSIQHIQFTLNHFAADVYVGPPKGNNWFEKQTAGTI     360
PELAEZ_DN3148_c0_g1_i11        FPLLVSFLPNWTERVLFVLASFCVCAIQHIQFTLNHFAADVYVGPPKGNNWFEKQTAGTI     360
SREEDHAR_DN6761_c0_g3_i4       FPLLVSFLPNWTERVLFVLASFCVCSIQHIQFTLNHFAADVYVGPPKGNNWFEKQTAGTI     360
                               *************************:**********************************

WIMBERLEY_DN1165_c0_g1_i4      DIDCPSYMDWFFGGLQFQLEHHLFPRLPRCHLRKVSPIIRDLCKKHNLPYRSLTFVEANK     420
GUPTA_DN2093_c1_g1_i9          DIDCPSYMDWFFGGLQFQLEHHLFPRLPR-------------------------------     389
PELAEZ_DN3148_c0_g1_i11        DIDCPSYMDWFFGGLQFQLEHHLFPRLPRCHLRKVSPIIRDLCKKHNLPYRSLTFVEANK     420
SREEDHAR_DN6761_c0_g3_i4       DIDCPSYMDWFFGGLQFQLEHHLFPRLPRCHLRKVSPIIRDLCKKHNLPYRSLTFVEANK     420
                               *****************************

WIMBERLEY_DN1165_c0_g1_i4      WTLRTLRAAAIEARDFSMVPRNLLWEAVNTHG 452
GUPTA_DN2093_c1_g1_i9          -------------------------------- 389
PELAEZ_DN3148_c0_g1_i11        WTLRTLRAAAIEARDFSMVPRNLLWEAVNTHG 452
SREEDHAR_DN6761_c0_g3_i4       WTLRTLRAAAIEARDFSMVPRNLLWEAVNTHG 452
```

Alignment 9: Sequences in this alignment bear high homology to A0A4D9BC00_SALSN: “Fatty acid desaturase 2 (Delta-6 desaturase).”

```
CLUSTAL O(1.2.4) multiple sequence alignment


WIMBERLEY_DN1165_c0_g1_i8      MAAAGDDDKKYITSDELSKHNKSGDLWLSIQGKVYNVTDWAKRHPGGEVPLLNLAGQDVT     60
GUPTA_DN2093_c1_g1_i10         MAAGGDDDKKYITSDELSKHNKSGDLWLSIQGKVYNVTDWAKRHPGGEVPLLNLAGQDVT     60
GUPTA_DN2093_c1_g1_i5          MAAGGDDDKKYITSDELSKHNKSGDLWLSIQGKVYNVTDWAKRHPGGEVPLLNLAGQDVT     60
PELAEZ_DN3148_c0_g1_i10        MAAAGDDDKKYITSDELSKHNKSGDLWLSIQGKVYNVTDWAKRHPGGEVPLLNLAGQDVT     60
SREEDHAR_DN6761_c0_g3_i2       MAAAGDDDKKYITSDELSKHNKSGDLWLSIQGKVYNVTDWAKRHPGGEVPLLNLAGQDVT     60
                               ***.********************************************************

WIMBERLEY_DN1165_c0_g1_i8      DAFIAFHPGSAWKHLDNFFTGYHLRDFHVSEMSRDYRTLATQFARSGMFEKKGHGVIYSL     120
GUPTA_DN2093_c1_g1_i10         DAFIAFHPGSAWKHLDSLFTGYHLRDFHVSEMSRDYRTLATQFARSGMFEKKGHGVIYSL     120
GUPTA_DN2093_c1_g1_i5          DAFIAFHPGSAWKHLDSLFTGYHLRDFHVSEMSRDYRTLATQFARSGMFEKKGHGVIYSL     120
PELAEZ_DN3148_c0_g1_i10        DAFIAFHPGSAWKHLDSLFTGYHLRDFHVSEMSRDYRTLATQFARSGMFEKKGHGVIYSL     120
SREEDHAR_DN6761_c0_g3_i2       DAFIAFHPGSAWKHLDSLFTGYHLRDFHVSEMSRDYRTLATQFARSGMFEKKGHGVIYSL     120
                               ****************.:******************************************

WIMBERLEY_DN1165_c0_g1_i8      CFVSFLLAACFYGVLGCDGFLTHILSGGLLGLIWMQVAYLGHDSGHYNIMISPRFNKLAQ     180
GUPTA_DN2093_c1_g1_i10         CFVSFLLAACFYGVLRCDGFLTHILSGGLLGLIWMQVAYLGHDSGHYNIMISPRFNKLAQ     180
GUPTA_DN2093_c1_g1_i5          CFVSFLLAACFYGVLRCDGFLTHILSGGLLGLIWMQVAYLGHDSGHYNIMISPRFNKLAQ     180
PELAEZ_DN3148_c0_g1_i10        CFISVLLAACFYGVLRCDGFLTHILSGGLLGLIWMQVAYLGHDSGHYNIMISPRFNKLAQ     180
SREEDHAR_DN6761_c0_g3_i2       CFISVLLAACFYGVLRCDGFLTHILSGGLLGLIWMQVAYLGHDSGHYNIMISPRFNKLAQ     180
                               **:*.********** ********************************************

WIMBERLEY_DN1165_c0_g1_i8      ILTGNCLTGISIAWWKWTHNAHHIACNSLDYDPDLQHLPMLAVSTRFFTNLTSKFYNRKL     240
GUPTA_DN2093_c1_g1_i10         ILTGNCLTGISIAWWKWTHNAHHIACNSLDYDPDLQHLPMLAVSTRFFTNLTSKFYNRKL     240
GUPTA_DN2093_c1_g1_i5          ILTGNCLTGISIAWWKWTHNAHHIACNSLDYDPDLQHLPMLAVSTRFFTNLTSKFYNRKL     240
PELAEZ_DN3148_c0_g1_i10        ILTGNCLTGISIAWWKWTHNAHHIACNSLDYDPDLQHLPMLAVSTRFFTNLTSKFYNRKL     240
SREEDHAR_DN6761_c0_g3_i2       ILTGNCLTGISIAWWKWTHNAHHIACNSLDYDPDLQHLPMLAVSTRFFTNLTSKFYNRKL     240
                               ************************************************************

WIMBERLEY_DN1165_c0_g1_i8      EFDPIARFFISYQHLTYYPVMCVARVNLYLQTFLLLLSNRRVPDRALNILGIMIFWTWFP     300
GUPTA_DN2093_c1_g1_i10         EFDPIARFFISYQHLTYYPVMCVARVNLYLQTFLLLLSNRRVPDRALNILGIMIFWTWFP     300
GUPTA_DN2093_c1_g1_i5          EFDPIARFFISYQHLTYYPVMCVARVNLYLQTFLLLLSNRRVPDRALNILGIMIFWTWFP     300
PELAEZ_DN3148_c0_g1_i10        EFDPIARFFISYQHLTYYPVMCVARVNLYLQTFLLLLSNRRVPDRALNILGIMIFWTWFP     300
SREEDHAR_DN6761_c0_g3_i2       EFDPIARFFISYQHLTYYPVMCVARVNLYLQTFLLLLSNRRVPDRALNILGIMIFWTWFP     300
                               ************************************************************

WIMBERLEY_DN1165_c0_g1_i8      LLVSCLPNWTERVLFVLASFCVCAIQHIQFTLNHFAADVYVGPPKGNNWFEKQTAGTIDI     360
GUPTA_DN2093_c1_g1_i10         LLVSCLPNWTERVLFVLASFCVCSIQHIQFTLNHFAADVYVGPPKGNNWFEKQTAGTIDI     360
GUPTA_DN2093_c1_g1_i5          LLVSCLPNWTERVLFVLASFCVCSIQHIQFTLNHFAADVYVGPPKGNNWFEKQTAGTIDI     360
PELAEZ_DN3148_c0_g1_i10        LLVSCLPNWTERVLFVLASFCVCAIQHIQFTLNHFAADVYVGPPKGNNWFEKQTAGTIDI     360
SREEDHAR_DN6761_c0_g3_i2       LLVSCLPNWTERVLFVLASFCVCSIQHIQFTLNHFAADVYVGPPKGNNWFEKQTAGTIDI     360
                               ***********************:************************************

WIMBERLEY_DN1165_c0_g1_i8      DCPSWMDWFFGGLQFQLEHHLFPRLPRCHLRKVSPIIRDLCKKHNLPYRSLTFVEANKWT     420
GUPTA_DN2093_c1_g1_i10         DCPSWMDWFFGGLQFQLEHHLFPRLPRCHLRKVSPIIRDLCKKHNLPYRSLTFVEANKWT     420
GUPTA_DN2093_c1_g1_i5          DCPSWMDWFFGGLQFQLEHHLFPRLPRCHLRKVSPIIRDLCKKHNLPYRSLTFVEANKWT     420
PELAEZ_DN3148_c0_g1_i10        DCPSWMDWFFGGLQFQLEHHLFPRLPRCHLRKVSPIIRDLCKKHNLPYRSLTFVEANKWT     420
SREEDHAR_DN6761_c0_g3_i2       DCPSWMDWFFGGLQFQLEHHLFPRLPRCHLRKVSPIIRDLCKKHNLPYRSLTFVEANKWT     420
                               ************************************************************

WIMBERLEY_DN1165_c0_g1_i8      LRTLRTAAVEARDFSMAPRNLLWEAVNTHG   450
GUPTA_DN2093_c1_g1_i10         LRTLRTAAVEARDFSMAPRNLLWEAVNTHG   450
GUPTA_DN2093_c1_g1_i5          LRTLRTAAVEARDFSMAPRNLLWEAVNTHG   450
PELAEZ_DN3148_c0_g1_i10        LRTLRTAAVEARDFSMAPRNLLWEAVNTHG   450
SREEDHAR_DN6761_c0_g3_i2       LRTLRTAAVEARDFSMAPRNLLWEAVNTHG   450
                               ******************************
```

Alignment 10: Sequences in this alignment bear homology to A0A1Z1EC60_9LAMI: “Fatty acid desaturase 3 isoform 2.” Once again, the *S. hispanica* sequence for this enzyme is already present in the UniProt KnowledgeBase.

```
CLUSTAL O(1.2.4) multiple sequence alignment


PELAEZ_DN10080_c0_g1_i3          MAVSSGARLSESGAEGGEPYAGQCEHLEGIGKRAADKFDPAAPPPFKIADIRAAIPPHCW    60
SREEDHAR_DN534_c0_g1_i15         MAVSSGARLSESGAEGGEPYAGQCEHLEGIGKRAADKFDPAAPPPFKIADIRAAIPPHCW    60
GUPTA_DN142_c1_g1_i3             MAVSSGADAEHH----------GHAQYEHLGKRAADKFDPAAPPPFKIADIRAAIPPHCW    50
SREEDHAR_DN534_c0_g1_i1          MAVSSGADAEHH----------GHAQYEHLGKRAADKFDPAAPPPFKIADIRAAIPPHCW    50
WIMBERLEY_DN4231_c0_g1_i12       MAVSSGADAEHH----------GHAQYEHLGKRAADKFDPAAPPPFKIADIRAAIPPHCW    50
                                 *******  ..              : * :******************************

PELAEZ_DN10080_c0_g1_i3          VKDPLRSLSYVAWDLIAVAALLAAAAFFDSWIFWPIYWAAQGTMFWALFVLGHDCGHGSF    120
SREEDHAR_DN534_c0_g1_i15         VKDPLRSLSYVAWDVFVVAALLAAAAFFDSWIFWPIYWAAQGTMFWALFVLGHDCGHGSF    120
GUPTA_DN142_c1_g1_i3             VKDPLRSLSYVAWDVFVVAALLAAAAFFDSWIFWPIYWAAQGTMFWALFVLGHDCGHGSF    110
SREEDHAR_DN534_c0_g1_i1          VKDPLRSLSYVAWDVFVVAALLAAAAFFDSWIFWPIYWAAQGTMFWALFVLGHDCGHGSF    110
WIMBERLEY_DN4231_c0_g1_i12       VKDPLRSLSYVAWDLIAVAALLAAAAFFDSWIFWPIYWAAQGTMFWALFVLGHDCGHGSF    110
                                 **************::.*******************************************

PELAEZ_DN10080_c0_g1_i3          SDNTTLNNVVGHVLHSSILVPYHGWRISHRTHHQNHGHVENDESWVPLTENLYKQLDFST    180
SREEDHAR_DN534_c0_g1_i15         SDNTTLNNVVGHVLHSSILVPYHGWRISHRTHHQNHGHVENDESWVPLTENLYKQLDFST    180
GUPTA_DN142_c1_g1_i3             SDNTTLNNVVGHVLHSSILVPYHGWRISHRTHHQNHGHVENDESWVPLTENLYKQLDFST    170
SREEDHAR_DN534_c0_g1_i1          SDNTTLNNVVGHVLHSSILVPYHGWRISHRTHHQNHGHVENDESWVPLTENLYKQLDFST    170
WIMBERLEY_DN4231_c0_g1_i12       SDNTTLNNVVGHVLHSSILVPYHGWRISHRTHHQNHGHVENDESWVPLTENLYKQLDFST    170
                                 ************************************************************

PELAEZ_DN10080_c0_g1_i3          KFLRYKIPFPMFAYPLYLWYRSPGKSGSHFNPYSSLFKPNERDLVITSTICWAAMVACLL    240
SREEDHAR_DN534_c0_g1_i15         KFLRYKIPFPMFAYPLYLWYRSPGKSGSHFNPYSSLFKPNERDLVITSTICWAAMVACLL    240
GUPTA_DN142_c1_g1_i3             KFLRYKIPFPMFAYPLYLWYRSPGKSGSHFNPYSSLFKPNERDLVITSTICWAAMVACLL    230
SREEDHAR_DN534_c0_g1_i1          KFLRYKIPFPMFAYPLYLWYRSPGKSGSHFNPYSSLFKPNERDLVITSTICWAAMVACLL    230
WIMBERLEY_DN4231_c0_g1_i12       KFLRYKIPFPMFAYPLYLWYRSPGKSGSHFNPYSSLFKPNERDLVITSTICWAAMVACLL    230
                                 ************************************************************

PELAEZ_DN10080_c0_g1_i3          YASTIVGPTMLFKLYGVPYLIFVVWLDTVTYLHHHGYDKKLPWYRSKEWSYLRGGLTTVD    300
SREEDHAR_DN534_c0_g1_i15         YASTIVGPTMLFKLYGVPYLIFVVWLDTVTYLHHHGYDKKLPWYRSKEWSYLRGGLTTVD    300
GUPTA_DN142_c1_g1_i3             YASTIVGPTMLFKLYGVPYLIFVVWLDTVTYLHHHGYDKKLPWYRSKEWSYLRGGLTTVD    290
SREEDHAR_DN534_c0_g1_i1          YASTIVGPTMLFKLYGVPYLIFVVWLDTVTYLHHHGYDKKLPWYRSKEWSYLRGGLTTVD    290
WIMBERLEY_DN4231_c0_g1_i12       YASTIVGPTMLFKLYGVPYLIFVVWLDTVTYLHHHGYDKKLPWYRSKEWSYLRGGLTTVD    290
                                 ************************************************************

PELAEZ_DN10080_c0_g1_i3          QDYGIFNKIHHDIGTHVVHHLFPQIPHYHLVEATREAKRVLGNYYREPRKSGAVPFHLVP    360
SREEDHAR_DN534_c0_g1_i15         QDYGIFNKIHHDIGTHVVHHLFPQIPHYHLVEATREAKRVLGNYYREPRKSGAVPFHLVP    360
GUPTA_DN142_c1_g1_i3             QDYGIFNKIHHDIGTHVVHHLFPQIPHYHLVEATREAKRVLGNYYREPRKSGAVPFHLVP    350
SREEDHAR_DN534_c0_g1_i1          QDYGIFNKIHHDIGTHVVHHLFPQIPHYHLVEATREAKRVLGNYYREPRKSGAVPFHLVP    350
WIMBERLEY_DN4231_c0_g1_i12       QDYGIFNKIHHDIGTHVVHHLFPQIPHYHLVEATREAKRVLGNYYREPRKSGAVPFHLVP    350
                                 ************************************************************

PELAEZ_DN10080_c0_g1_i3          TLLKSLSRDHYVSDNGDIVYYQTDGELFSSKEI    393
SREEDHAR_DN534_c0_g1_i15         TLLKSLSRDHYVSDNGDIVYYQTDGELFSSKEI    393
GUPTA_DN142_c1_g1_i3             TLLKSLSRDHYVSDNGDIVYYQTDGELFSSKEI    383
SREEDHAR_DN534_c0_g1_i1          TLLKSLSRDHYVSDNGDIVYYQTDGELFSSKEI    383
WIMBERLEY_DN4231_c0_g1_i12       TLLKSLSRDHYVSDNGDIVYYQTDGELFSSKEI    383
                                 *********************************
```
